# The *Klebsiella pneumoniae* citrate synthase gene, *gltA*, influences site specific fitness during infection

**DOI:** 10.1371/journal.ppat.1008010

**Published:** 2019-08-26

**Authors:** Jay Vornhagen, Yuang Sun, Paul Breen, Valerie Forsyth, Lili Zhao, Harry L. T. Mobley, Michael A. Bachman

**Affiliations:** 1 Department of Pathology, University of Michigan, Ann Arbor, United States of America; 2 Department of Microbiology & Immunology, University of Michigan, Ann Arbor, United States of America; 3 Department of Biostatistics, School of Public Health, University of Michigan, Ann Arbor, United States of America; University of North Carolina at Chapel Hil, UNITED STATES

## Abstract

*Klebsiella pneumoniae* (Kp), one of the most common causes of healthcare-associated infections, increases patient morbidity, mortality, and hospitalization costs. Kp must acquire nutrients from the host for successful infection; however, the host is able to prevent bacterial nutrient acquisition through multiple systems. This includes the innate immune protein lipocalin 2 (Lcn2), which prevents Kp iron acquisition. To identify novel Lcn2-dependent Kp factors that mediate evasion of nutritional immunity during lung infection, we undertook an InSeq study using a pool of >20,000 transposon mutants administered to *Lcn2*^*+/+*^ and *Lcn2*^*-/-*^ mice. Comparing transposon mutant frequencies between mouse genotypes, we identified the Kp citrate synthase, GltA, as potentially interacting with Lcn2, and this novel finding was independently validated. Interestingly, *in vitro* studies suggest that this interaction is not direct. Given that GltA is involved in oxidative metabolism, we screened the ability of this mutant to use a variety of carbon and nitrogen sources. The results indicated that the *gltA* mutant has a distinct amino acid auxotrophy rendering it reliant upon glutamate family amino acids for growth. Deletion of *Lcn2* from the host leads to increased amino acid levels in bronchioloalveolar lavage fluid, corresponding to increased fitness of the *gltA* mutant *in vivo* and *ex vivo*. Accordingly, addition of glutamate family amino acids to *Lcn2*^*+/+*^ bronchioloalveolar lavage fluid rescued growth of the *gltA* mutant. Using a variety of mouse models of infection, we show that GltA is an organ-specific fitness factor required for complete fitness in the spleen, liver, and gut, but dispensable in the bloodstream. Similar to bronchioloalveolar lavage fluid, addition of glutamate family amino acids to *Lcn2*^*+/+*^ organ lysates was sufficient to rescue the loss of *gltA*. Together, this study describes a critical role for GltA in Kp infection and provides unique insight into how metabolic flexibility impacts bacterial fitness during infection.

## Author summary

The bacteria *Klebsiella pneumoniae* (Kp) is an important cause of infection in healthcare settings. These infections can be difficult to treat, as they frequently occur in chronically ill patients and the bacteria have the ability to acquire multiple antibiotic resistance markers. Kp is a common colonizer of the intestinal tract in hospitalized patients, and can progress to infections of the bloodstream, respiratory, and urinary tract. However, the bacterial factors that allow Kp to replicate in these different body sites are unclear. In this study, we found that the Kp citrate synthase, GltA, enables bacterial replication in the lung and intestine by enhancing the ability of Kp to use diverse nutrients in a mechanism known as metabolic flexibility. Kp lacking GltA require specific amino acids that are abundant in blood, but not other body sites. The work in this study provides novel insight into why Kp is a successful hospital pathogen that can colonize and infect multiple body sites.

## Introduction

Extended spectrum beta-lactamase (ESBL)-producing *Enterobacteriaceae* and carbapenem-resistant *Enterobacteriaceae* (CRE) pose a serious public health threat due to their extensive antibiotic resistance. Many ESBL and CRE infections are healthcare-associated infections (HAIs), meaning they occur in long-term healthcare facilities and hospitals. *Klebsiella pneumoniae* (Kp) is an environmentally ubiquitous member of the *Enterobacteriaceae* family that can acquire antibiotic resistance genes [1], and thus is a leading cause of ESBL-producing *Enterobacteriaceae* infections [2] and HAIs [3]. Mortality rates in patients infected with antibiotic resistant Kp often exceed 40% [4]. Disturbingly, reports of hypervirulent clones of Kp acquiring mobile antibiotic resistance genes are becoming more frequent [5,6], posing a significant global threat to human health. As the efficacy of antibiotics diminishes and therapeutic options for patients infected with antibiotic resistant strains of Kp become increasingly limited, a better understanding of how Kp establish productive infections is necessary for the development of novel diagnostics and interventions to combat these dangerous bacteria.

To establish a productive infection, bacterial pathogens such as Kp must acquire nutrients from the host environment. Subsequently, metabolic flexibility dictates the capacity of pathogens to invade different niches [7–10]. This flexibility is defined by the ability of a bacterial pathogen to acquire and utilize different metabolites. For example, *Salmonella enterica* serotype Typhimurium uses tetrathionate as an electron acceptor, providing a fitness advantage in the gut, whereas this advantage is not conferred in the spleen due to the lack of tetrathionate [11]. Interestingly, Kp potentially exhibits diversity in metabolism and nutrient acquisition, as indicated by the ability to cause a wide range of severe infections, including pneumonia, bacteremia, urinary tract infection, and pyogenic liver abscess [12]. Additionally, infectious Kp frequently originates from sites of colonization [13–15], including the gut and nasopharynx [16,17]; however, the impact of metabolic flexibility on Kp pathogenesis has not received significant attention.

Metabolites necessary for niche invasion by pathogens can be acquired directly from the host, through the metabolic activity of other microorganisms, or by *de novo* synthesis. Limitation of access to these nutrients by the host is a universal means of impeding niche invasion by bacterial pathogens [18–21]. For example, iron is critical for niche invasion and subsequent pathogenesis, and thus the host actively sequesters iron from invading bacteria [18]. To overcome this nutrient limitation, pathogens such as Kp encode a variety of proteins and small molecules to harvest sequestered iron, including the family of low molecular weight chelators known as siderophores [22]. Consequently, the host further prevents iron acquisition by sequestering bacterial siderophores with the innate immune molecule Lipocalin 2 (Lcn2) [23]. In turn, Kp circumvents Lcn2 activity by expressing alternative siderophores [24]. Interestingly, Lcn2 has immunomodulatory effects [25], suggesting that the impact of Lcn2 during Kp infection is not limited to sequestration of iron and that there are likely other Lcn2-dependent Kp factors; however, this has yet to be experimentally addressed.

To discover Kp genes that are conditionally essential in the presence of Lcn2, we undertook an InSeq experiment comparing lung infection in *Lcn2*^*+/+*^ and *Lcn2*^*-/-*^ murine backgrounds. This revealed multiple conditionally essential genes, including the citrate (Si)-synthase gene *gltA*. *In vitro* studies indicated that the interaction between GltA and Lcn2 is indirect. Further analysis revealed that deletion of *gltA* dramatically reduces metabolic flexibility, leading to glutamate family amino acid auxotrophy and a severe limitation of glycolytic substrate utilization. This limitation of metabolic flexibility is partially complemented during lung infection by deletion of *Lcn2*, corresponding to an increase in glutamate family amino acid concentrations in the lung. Addition of these amino acids to growth media and bronchoalveolar fluid is sufficient to restore the loss of GltA. Using multiple murine models of infection, we show that GltA is not necessary for blood infection, but is necessary in the spleen, liver, and gut. Together, these data reveal how Kp metabolic flexibility, conferred by the citrate (Si)-synthase GltA, impacts site-specific fitness during infection.

## Results

To comprehensively identify novel Lcn2-dependent Kp factors during lung infection, we employed a previously described transposon library in the Kp strain KPPR1 [26,27]. To this end, *Lcn2*^*+/+*^ and *Lcn2*^*-/-*^ mice [28] were retropharyngeally inoculated with a pool of ~25,000 transposon mutants (S1 Fig). Twenty-four hours after inoculation total lung CFU were collected for DNA extraction and InSeq analysis, as previously described [26]. After filtering, each sample had greater than 50 million reads corresponding to greater than 20,000 unique transposon insertions inside of open reading frames (S1 Data). To identify Lcn2-dependent Kp factors, the ratio of transposon insertion reads within each gene between the *Lcn2*^*+/+*^ and *Lcn2*^*-/-*^ lung output pools (mean reads per gene were 169 and 156, respectively) was calculated to generate a fitness index. Over 1,600 genes were significantly enriched in either mouse background indicating a potential interaction with Lcn2, including *entB*, which is required to synthesize both enterobactin and the Lcn2-evading siderophore salmochelin (S1 Data). To limit our analysis, only genes with a significant fitness index greater or less than 3 standard deviations from the mean were considered Lcn2-dependent, leading to a final list of 43 candidate genes (Table 1, S1 Fig, S1 Data).

**Table 1:** Lcn2-dependent *K*. *pneumoniae* factors.

Locus ID (VK055_#)	Gene Name	Log_10_ Ratio (*Lcn2*^*+/+*^: *Lcn2*^*-/-*^)	*P* Value	GenBank Definition
2967	*phnG*	-2.27	5.82E-11	phosphonate C-P lyase system protein PhnG
2193	* *	-2.24	2.84E-14	glyoxalase
1802	*gltA*	-2.23	0.00E+00	citrate (Si)-synthase
358	* *	-2.14	4.44E-16	putative siderophore transport system ATP-binding protein YusV
852	* *	-2.08	1.39E-17	diguanylate cyclase domain protein
4742	* *	-1.97	7.94E-21	hypothetical protein
1264	* *	-1.91	3.56E-266	putative enzyme related to aldose 1-epimerase
679	*pqqB*	-1.89	1.46E-11	coenzyme PQQ biosynthesis protein B
4503	* *	-1.88	7.45E-09	hypothetical protein
3425	* *	-1.80	9.31E-10	putative efflux pump outer membrane protein TtgC
2801	* *	-1.76	0.00E+00	bacterial regulatory, GntR family protein
406	*budC*	-1.76	0.00E+00	diacetyl reductase (S-acetoin forming)
1665	*nikE*	-1.74	2.98E-08	nickel import ATP-binding protein NikE2
494	* *	-1.73	9.41E-30	efflux transporter, RND family, MFP subunit
4907	* *	-1.72	4.07E-09	yejG-like family protein
4402	*cysD*	-1.71	0.00E+00	CysD
4417	* *	-1.71	9.54E-07	MarR family protein
23	*rcsA*	-1.68	3.82E-11	transcriptional regulator of capsular polysaccharide synthesis
3697	*envZ*	-1.66	0.00E+00	EnvZ
1081	* *	-1.64	7.63E-06	bacterial regulatory helix-turn-helix, LysR family protein
2100	* *	-1.64	4.62E-14	primosomal replication PriB and PriC family protein
5166	* *	-1.56	5.12E-12	hypothetical protein
5008	*udk*	-1.55	0.00E+00	deoxycytidine triphosphate deaminase
1530	* *	-1.55	2.84E-10	hypothetical protein
2620	* *	-1.54	1.08E-09	hypothetical protein
4957	* *	-1.54	1.14E-80	osmoprotectant transport system ATP-binding protein
2890	* *	-1.49	4.44E-37	hypothetical protein
2734	* *	1.68	3.46E-103	hypothetical protein
3005	*soxR*	1.69	1.54E-08	redox-sensitive transcriptional activator SoxR
631	* *	1.70	4.95E-23	zinc-binding dehydrogenase family protein
503	* *	1.74	3.82E-11	pyridoxal kinase
1089	* *	1.79	4.02E-21	type VI secretion lipofamily protein
2579	* *	1.80	9.59E-27	CreA family protein
1763	*galE*	1.90	2.91E-11	UDP-glucose 4-epimerase
3375	* *	1.91	1.20E-19	hypothetical protein
3418	* *	1.92	1.94E-55	PrpF family protein
3618	*nikC*	1.93	7.05E-18	nickel ABC transporter, permease subunit NikC
4369	* *	1.97	0.00E+00	binding-protein-dependent inner membrane transport system
2230	*iraP*	2.02	8.70E-46	anti-adaptor protein for sigmaS stabilization
2009	* *	2.04	1.57E-20	hypothetical protein
4943	* *	2.07	1.46E-11	OprB family protein
953	* *	2.33	5.55E-17	response regulator
1873	*ybeC*	2.45	3.55E-15	TatE

**Fig 1:**
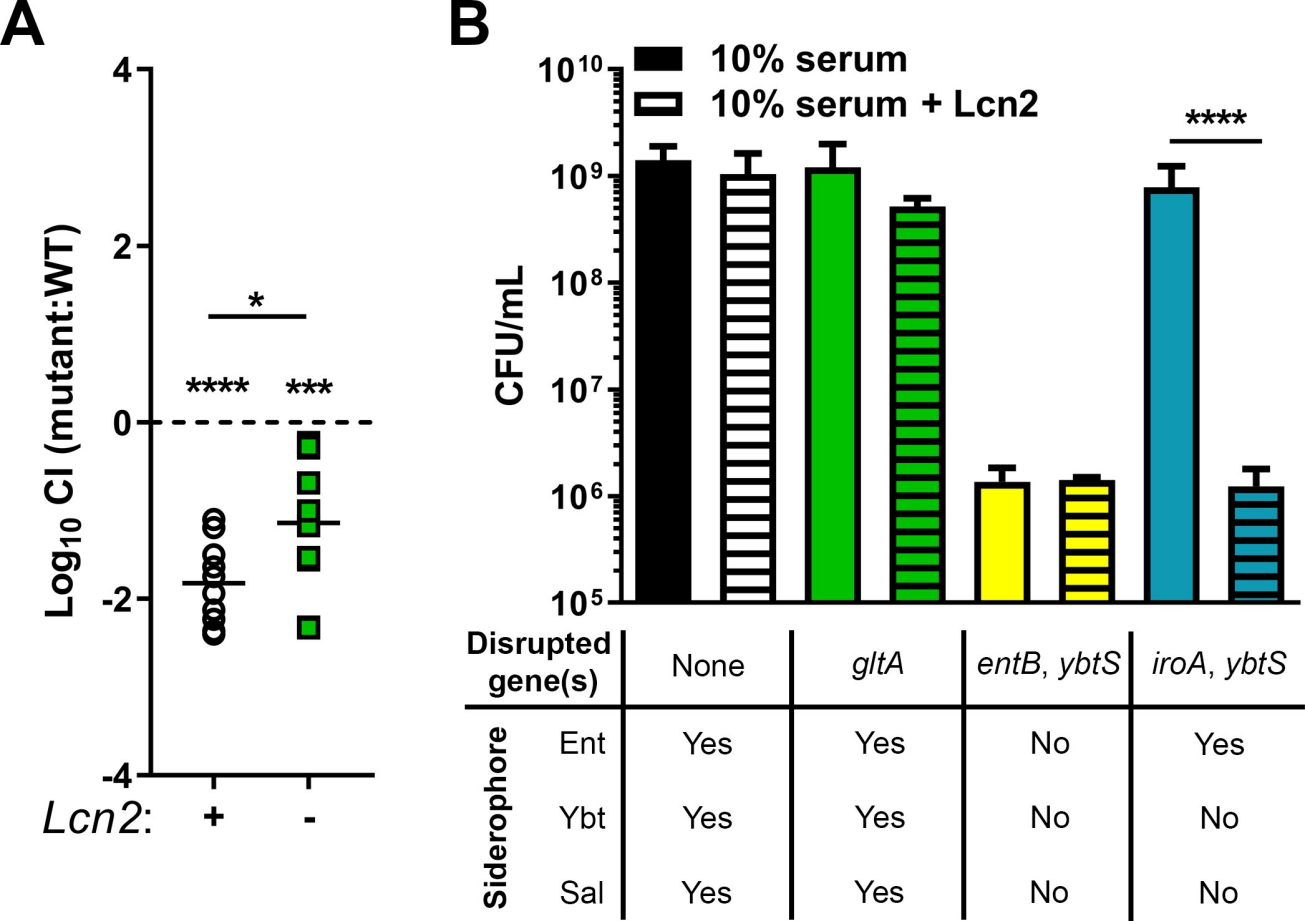
The Kp citrate synthase, *gltA*, interacts indirectly with Lcn2 during lung infection. (A) A *gltA* (VK055_1802) mutant was constructed and used to validate InSeq findings. C57BL/6J mice or isogenic *Lcn2*^*-/-*^ mice were retropharyngeally inoculated with approximately 1×10^6^ CFU of a 1:1 mix of WT KPPR1 and KPPR1Δ*gltA*. Lung bacterial burden was measured after 24 hours, and log_10_ competitive index of the mutant strain compared to the WT strain was calculated for each mouse strain (n = 10, mean displayed, *P < 0.05, ***P < 0.0005, ****P < 0.00005, one-sample *t* test or Student’s *t* test). (B) WT KPPR1 and various isogenic mutants were grown in RPMI + 10% (v/v) heat-inactivated resting human serum ± purified recombinant human Lcn2 overnight, then total CFU was enumerated by dilution plating on selective media (n = 3–4, mean displayed ± SEM, ****P < 0.00005, Student’s *t* test).

Sixteen transposon mutants displayed enhanced fitness when Lcn2 is present, and 27 displayed enhanced fitness when Lcn2 is absent (Table 1). Interestingly, the most common molecular function of genes enriched in both backgrounds was metabolism [29], accounting for 10 of 43 genes (23.3%, S1 Data). This was followed by membrane transport (6 of 43, 14.0%), including the putative siderophore transport system ATP-binding protein YusV, transcription factors (3 of 43, 7.0%), two-component systems (3 of 43, 6.9%), DNA repair and recombination (2 of 43, 4.7%), protein export (1 of 43, 2.3%), and quorum sensing (1 of 43, 2.3%). The molecular function of 17 (39.5%) of these genes has not been characterized. Three genes (VK055_1802, VK055_3697, and VK055_4417) enriched in the *Lcn2*^*-/-*^ background were previously identified as fitness factors during Kp lung infection [26] (S1 Data, in italics). Five genes displayed a greater than 100-fold enrichment in the *Lcn2*^*-/-*^ lung pool, and one, VK055_1802, had a *P* value less than 10^−300^ (Table 1, S1 Fig, S1 Data). VK055_1802 is annotated as *gltA*, which encodes the citric acid cycle enzyme citrate (Si)-synthase [30]. Together, these data indicate that metabolism is critical for the interaction between KPPR1 and Lcn2 during lung infection and that *gltA* is potentially a Lcn2-dependent metabolic Kp gene. To confirm the role of *gltA* during lung infection and its dependence on Lcn2, we constructed an isogenic *gltA* mutant [31] and complemented the gene *in trans*. The KPPR1Δ*gltA* strain displayed no growth defect compared to WT KPPR1 in nutrient-rich media (S1 Fig). This mutant was mixed 1:1 with its WT parent strain, then inoculated retropharyngeally in both *Lcn2*^*+/+*^ and *Lcn2*^*-/-*^ mice. As observed with InSeq data, the KPPR1Δ*gltA* mutant displayed a 67-fold mean fitness defect compared to the WT KPPR1 strain in the *Lcn2*^*+/+*^ lung that was partially alleviated in the *Lcn2*^*-/-*^ lung (Fig 1A, S2 Fig). Mono-infections in *Lcn2*^*+/+*^ mice also revealed a significant defect in growth of the KPPR1Δ*gltA* mutant (S2 Fig). Given that citrate can act as a weak siderophore [32] and is a building block of complex siderophores [33,34], we hypothesized that loss of siderophore activity through deletion of *gltA* may explain the observed loss of fitness. To test this hypothesis, we grew a variety of KPPR1-derived strains in RPMI plus 10% human serum, which is iron limited to Kp due to the activity of transferrin [35], with or without recombinant human Lcn2. The WT KPPR1 strain was not affected by the presence of Lcn2; however, the siderophore-null KPPR1Δ*entB*Δ*ybtS* strain [36] was unable to grow in Lcn2-free conditions, consistent with the importance of siderophore function for KPPR1 growth. The enterobactin-dependent KPPR1Δ*iroA*Δ*ybtS* strain [35] was able to grow in Lcn2-free conditions, but unable to grow in the presence of Lcn2, validating the antagonistic relationship between enterobactin and Lcn2 (Fig 1B). Deletion of *gltA* had no impact on growth in the presence of Lcn2 (Fig 1B), indicating that citrate produced by GltA is not involved in detectable siderophore activity and suggesting that the relationship between *gltA* and Lcn2 during lung infection is indirect.

**Fig 2:**
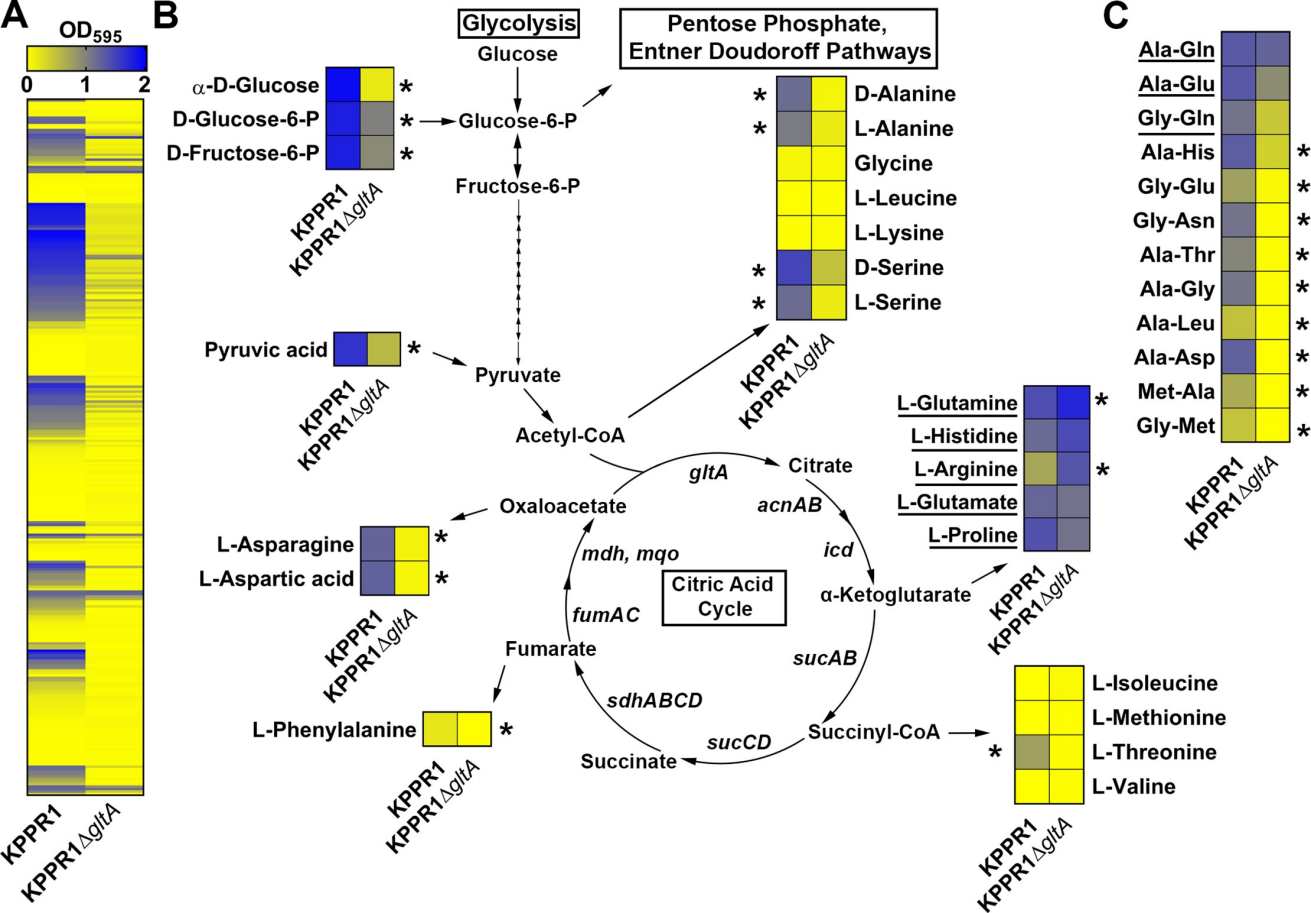
Deletion of *gltA* leads to diminished metabolic flexibility and distinct amino acid auxotrophy. (A) Heatmap summarizing BioLog Phenotype Microarray analysis of WT KPPR1 and KPPR1Δ*gltA* growth in 282 carbon and nitrogen limited growth conditions indicates multiple conditions that sustained growth of WT KPPR1, but not KPPR1Δ*gltA*. (B) A subset of growth conditions summarizing glycolysis and non-essential amino acid biosynthesis that indicates a distinct amino acid auxotrophy is induced by deletion of *gltA*. Arrows from citric acid cycle intermediates indicate amino acids that utilize these intermediates for biosynthesis. (C) A subset of growth conditions summarizing dipeptide utilization that further indicates induction of a distinct amino acid auxotrophy by deletion of *gltA* (n = 3, mean displayed, *P < 0.05, Student’s *t* test). Underlined substrates support equivalent or enhanced growth of KPPR1Δ*gltA* relative to WT KPPR1.

**Fig 3:**
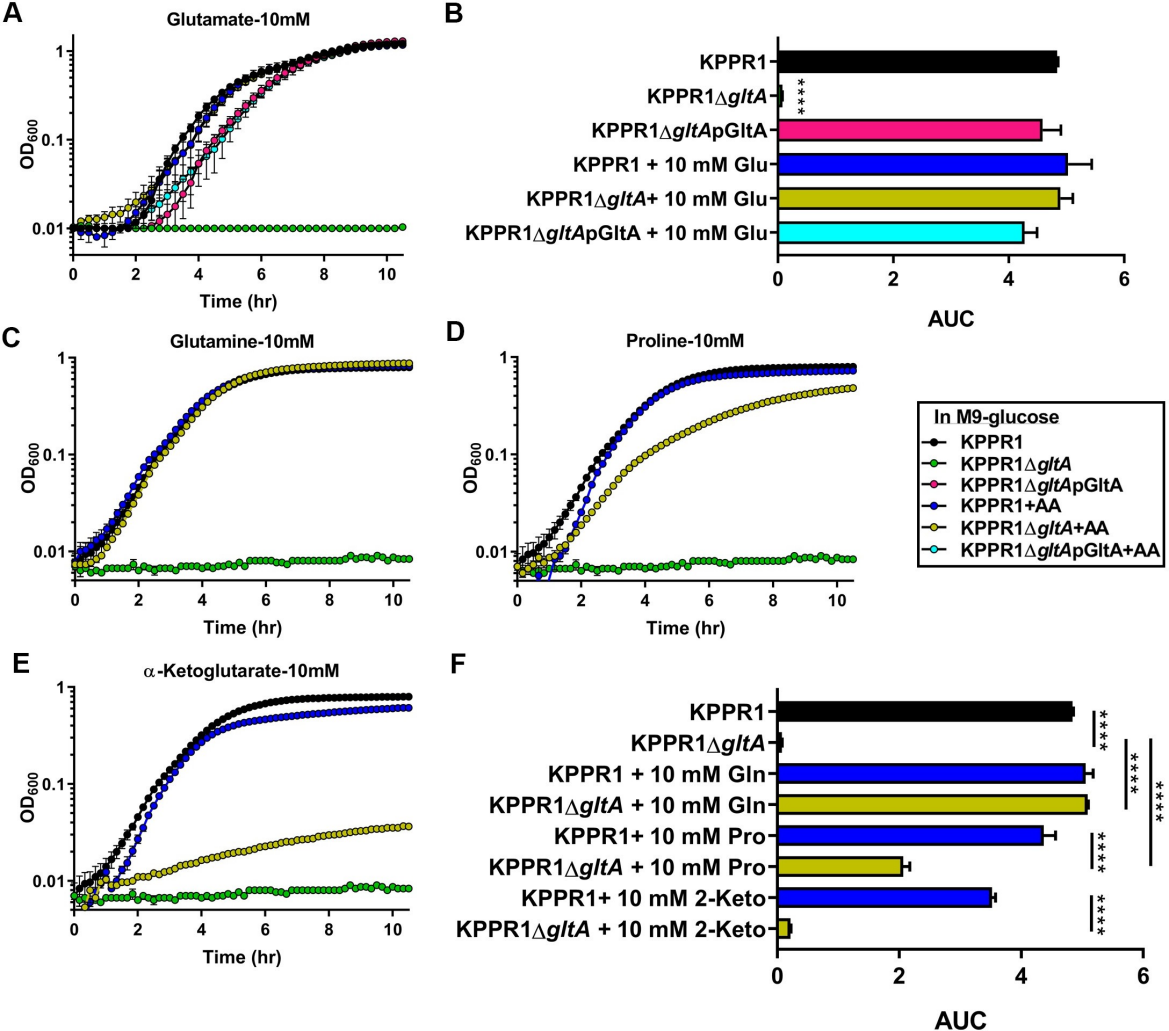
Auxotrophy due to deletion of *gltA* is functionally complemented by glutamate and glutamate family amino acids. (A) WT KPPR1, KPPR1Δ*gltA*, and KPPR1Δ*gltA*pGltA were grown in M9 minimal media + 0.4% glucose with or without 10 mM glutamate (n = 3, mean displayed ± SEM). (B) AUC analysis of WT KPPR1, KPPR1Δ*gltA*, and KPPR1Δ*gltA*pGltA growth in M9 minimal media +0.4% glucose with 10 mM glutamate (n = 3, ****P < 0.00005 compared to all other groups, Tukey’s multiple comparison test following ANOVA, mean displayed ± SEM). WT KPPR1 and KPPR1Δ*gltA* were grown in M9 minimal media + 0.4% glucose with 10 mM (C) glutamine, (D) proline, and (E) 2-α-ketoglutarate (n = 3, mean displayed ± SEM). “+AA” label indicates addition of amino acids to growth media at concentrations indicated in graph title. (F) AUC analysis of WT KPPR1 and KPPR1Δ*gltA* growth in M9 minimal media + 0.4% glucose + specific amino acid (n = 3, ***P < 0.0005, ****P < 0.00005, Tukey’s multiple comparison test following ANOVA, mean displayed ± SEM). Data presented in panels C-E were generated simultaneously, but graphed separately for ease of visualization.

Given that citrate (Si)-synthase performs an irreversible oxidation step in the citric acid cycle, we next postulated that the relationship between *gltA* and Lcn2 is due to disruption of the TCA cycle. In addition to oxidative metabolism, the TCA cycle provides a number of key carbon skeletons for the biosynthesis of amino acids. Growth of the KPPR1Δ*gltA* mutant was partially restored when citrate was provided as the sole carbon source as measured by area under curve (AUC) analysis [37,38] (S3 Fig) although both the WT and mutant grow slowly. Mutant growth was not restored when citrate is provided in combination with glucose (S3 Fig), as citrate is able to inhibit glycolysis through allosteric inhibition of phosphofructokinase [39].To determine if loss of GltA affects Kp metabolic capabilities, we performed an unbiased screen of carbon and nitrogen sources using the BioLog system to identify conditions differentially permissive to KPPR1Δ*gltA* growth [40]. Both strains were cultured under 282 different conditions, and growth was measured after 24 hours (Fig 2A, S2 Data). These experiments revealed 129 conditions in which WT KPPR1 significantly outgrew KPPR1Δ*gltA* and three conditions (L-arginine, L-glutamine, and ethylenediamine) in which KPPR1Δ*gltA* significantly outgrew WT KPPR1 (Fig 2A, S2 Data). Complete BioLog analysis revealed that deletion of *gltA* resulted in glutamate family amino acid (glutamate, glutamine, proline, histidine, and arginine) auxotrophy, as indicated by the ability of these amino acids to support growth of the KPPR1Δ*gltA* strain (Fig 2B, S2 Data). The glutamate family of amino acids is critical for multiple overlapping cellular functions, as these amino acids act as the juncture between glycolysis and gluconeogenesis [41], play an intermediary role in nitrogen assimilation [42], and act as a building block for peptidoglycan [43] and proteins (S4 Fig). Consistent with the finding that glutamate family amino acids complement the loss of GltA, some dipeptides containing these residues were partially or fully able to support growth of the KPPR1Δ*gltA* strain, whereas dipeptides without these residues did not (Fig 2C, S2 Data). To confirm our findings, WT KPPR1, KPPR1Δ*gltA*, and pGltA complemented strains were grown in minimal medium containing glucose. When glucose is the sole carbon source, the KPPR1Δ*gltA* strain is unable to grow (Fig 3A and 3B), and this phenotype was replicated for a variety of conditions wherein only a single sugar was provided as a sole carbon source (S5 Fig). The pGltA plasmid or addition of 10 mM glutamate fully restored growth of the KPPR1Δ*gltA* strain (Fig 3A and 3B) and growth was also fully or partially complemented by addition of 10 mM glutamine, proline, and α-ketoglutarate (Fig 3C–3F). Additionally, the restoration of KPPR1Δ*gltA* growth in M9 medium containing glucose by addition of glutamate is dose-dependent (S6 Fig). Together, these data indicate that deletion of *gltA* results in glutamate family amino acid auxotrophy.

**Fig 4:**
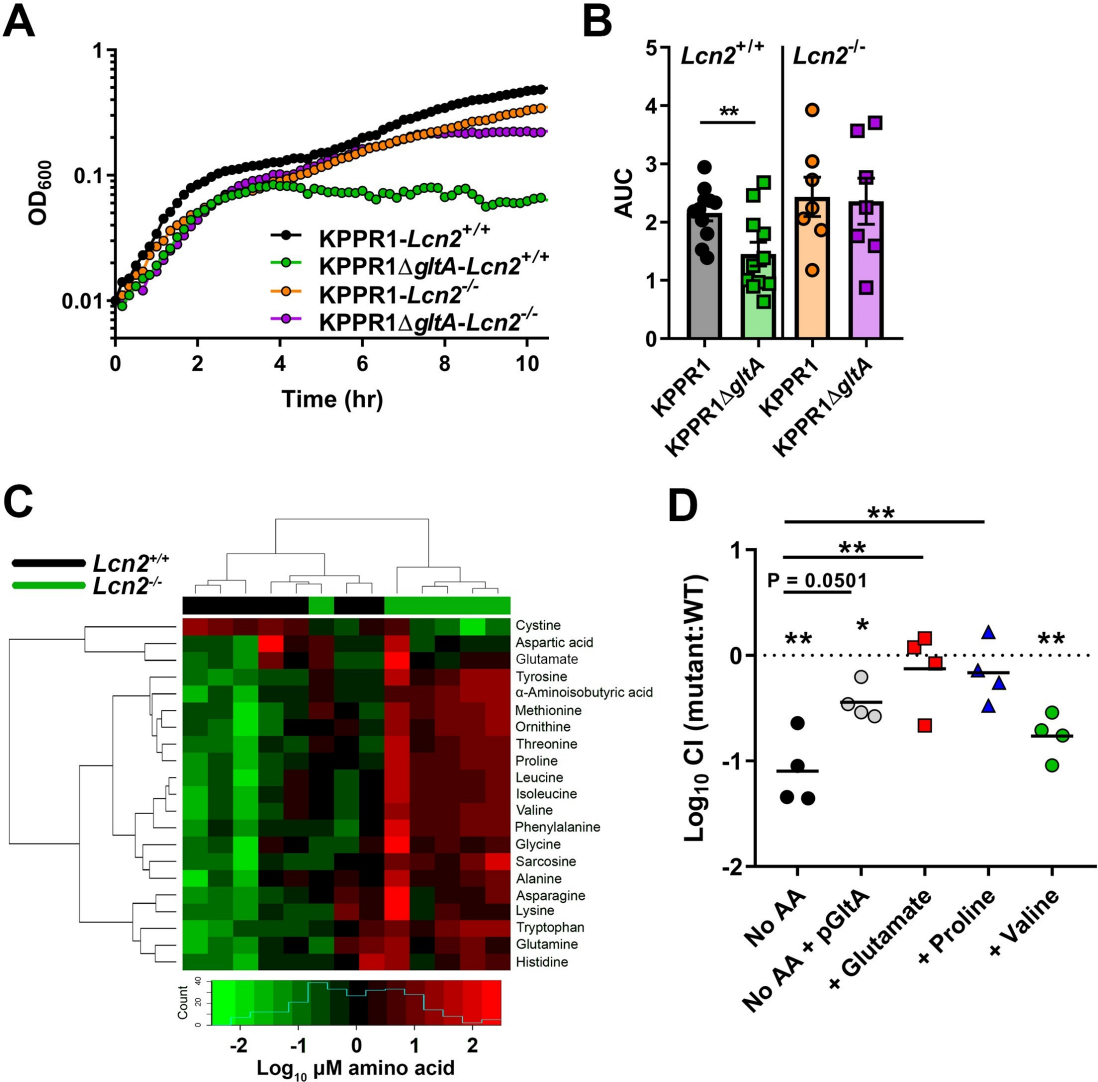
Bronchoalveolar lavage fluid from *Lcn2*^*-/-*^ mice can sustain growth of KPPR1Δ*gltA* due to increased amino acid levels. (A) Murine bronchoalveolar lavage fluid (BALF) was obtained from uninfected C57BL/6J mice or isogenic *Lcn2*^*-/-*^ mice, and WT KPPR1 and KPPR1Δ*gltA* were grown in BALF (representative curve displayed). (B) Area under curve (AUC) analysis was used to compare growth of WT KPPR1 and KPPR1Δ*gltA* in *Lcn2*^*+/+*^ and *Lcn2*^*-/-*^ BALF. (n = 7–11 per group, paired *t* test, mean displayed ± SEM, **P < 0.005). (C) Heatmap of amino acid concentrations in BALF obtained from uninfected C57BL/6J mice or isogenic *Lcn2*^*-/-*^ mice subjected to metabolomic analysis (n = 6–7 mice per group). Blue histogram in inset indicates composite amino acid concentration values in heatmap matrix. (D) Murine bronchoalveolar lavage fluid obtained from uninfected C57BL/6J mice with or without amino acids was inoculated with a 1:1 mix of WT KPPR1 and KPPR1Δ*gltA* or WT KPPR1 and KPPR1Δ*gltA*pGltA. Bacterial burden was measured after 24 hours, and log_10_ competitive index of the mutant strain compared to the WT strain was calculated for each sample (n = 4 per group, **P < 0.005, ***P < 0.0005, ****P < 0.00005, one-sample *t* test or Tukey’s multiple comparison test following ANOVA).

Airway lining fluid can be a nutritional source for bacteria living in the respiratory tract, with measurable levels of amino acids in both human and mouse bronchioloalveolar lavage fluid (BALF) [44–47]. To determine if the differences in KPPR1Δ*gltA* fitness in *Lcn2*^+/+^ and *Lcn2*^-/-^ lungs is attributable to differences in amino acid concentrations, whole BALF was collected from uninfected mice and used as a bacterial growth medium. BALF from both mouse strains sustained growth of WT KPPR1; however, KPPR1Δ*gltA* growth was defective in *Lcn2*^+/+^ BALF but not in *Lcn2*^-/-^ BALF (Fig 4A and 4B). To determine if there are inherent differences in amino acid levels in the lungs of *Lcn2*^+/+^ and *Lcn2*^-/-^ mice that explain these differences in growth, we used gas chromatography–mass spectrometry to measure free amino acid content in BALF from uninfected mice. *Lcn2*^-/-^ BALF contains significantly higher levels of multiple amino acids, including those in the glutamate family of amino acids, and higher total protein content (Table 2, S3 Data). Indeed, BALF from *Lcn2*^*+/+*^ and *Lcn2*^*-/-*^ are distinguishable based on their amino acid composition (Fig 4C). This increase in amino acid and protein content likely explains the difference between KPPR1Δ*gltA* fitness in the *Lcn2*^*+/+*^ and *Lcn2*^*-/-*^ backgrounds by functionally complementing the loss of *gltA*. To test this premise, KPPR1Δ*gltA* was mixed 1:1 with its WT parent strain, then inoculated in BALF from *Lcn2*^*+/+*^ mice. Addition of glutamate and proline to *Lcn2*^*+/+*^ BALF was sufficient to restore the fitness of the KPPR1Δ*gltA* strain, as was *in trans* complementation of GltA (Fig 4D). To assess if this effect was specific to glutamate family amino acids, we tested the ability of valine to restore the fitness of the KPPR1Δ*gltA* strain. Although its concentration was significantly higher in *Lcn2*^*-/-*^ BALF compared to *Lcn2*^*+/+*^ BALF (Table 2, Fig 4C, S3 Data), addition of valine was unable to restore the fitness of the KPPR1Δ*gltA* strain (Fig 4D). Together, these data indicate that the loss of fitness observed in the *Lcn2*^*+/+*^ lung is due to a reduction in metabolic flexibility induced by *gltA* deletion, and the increased concentration of glutamate family amino acids in the *Lcn2*^*-/-*^ background rescues the loss of *gltA*.

**Table 2:** Summary of amino acid and protein quantification from *Lcn2*^*+/+*^ and *Lcn2*^*-/-*^ BALF.

Metabolite*	*Lcn2*^*+/+*^ BALF (mean ± S.D.)	*Lcn2*^*-/-*^ BALF (mean ± S.D.)	Fold change	*P* Value	Adjusted *P* Value
Total protein	275 ± 100	457 ± 146	1.66	0.0513	-
Total free amino acids	276 ± 66.2	405 ± 93.6	1.47	**0.0221**	-
Alanine	31.4 ± 10.6	48.6 ± 11.9	1.55	**0.0350**	**0.0186**
Alpha-aminoisobutyric acid	0.22 ± 0.05	0.46 ± 0.06	2.03	**0.0012**	**0.0019**
Asparagine	6.54 ± 1.07	8.47 ± 2.09	1.29	0.0734	**0.0346**
Aspartic acid	11.7 ± 6.73	12.6 ± 4.53	1.08	0.2949	0.1123
Cystine	1.03 ± 0.45	0.34 ± 0.28	0.34	**0.0082**	**0.0054**
Glutamate	9.85 ± 3.86	16.7 ± 9.87	1.70	**0.0350**	**0.0186**
Glutamine	74.1 ± 25.2	95.3 ± 24.2	1.29	0.1807	0.0723
Glycine	43.4 ± 8.55	55.9 ± 12.9	1.29	0.0513	**0.0256**
Histidine	8.59 ± 2.37	10.3 ± 1.84	1.21	0.1375	0.0579
Isoleucine	5.89 ± 1.69	10.1 ± 1.98	1.73	**0.0047**	**0.0037**
Leucine	11.2 ± 3.01	19.7 ± 4.10	1.76	**0.0023**	**0.0023**
Lysine	19.3 ± 4.92	25.4 ± 8.81	1.31	0.1375	0.0579
Methionine	3.99 ± 1.19	9.64 ± 1.42	2.41	**0.0012**	**0.0019**
Ornithine	3.27 ± 1.18	6.77 ± 1.73	2.07	**0.0047**	**0.0037**
Phenylalanine	7.95 ± 1.19	13.0 ± 3.04	1.64	**0.0023**	**0.0023**
Proline	5.95 ± 1.37	11.8 ± 2.42	2.00	**0.0012**	**0.0019**
Sarcosine	0.23 ± 0.04	0.35 ± 0.08	1.51	**0.0140**	**0.0086**
Threonine	9.35 ± 2.07	17.6 ± 4.02	1.89	**0.0012**	**0.0019**
Tyrosine	4.78 ± 0.83	9.87 ± 2.01	2.06	**0.0012**	**0.0019**
Tryptophan	3.73 ± 1.48	6.92 ± 1.91	1.85	**0.0082**	**0.0054**
Valine	13.6 ± 4.20	25.4 ± 3.96	1.86	**0.0023**	**0.0023**

*Total protein concentration in μg/mL, all amino acid concentrations in μM

**Fig 5:**
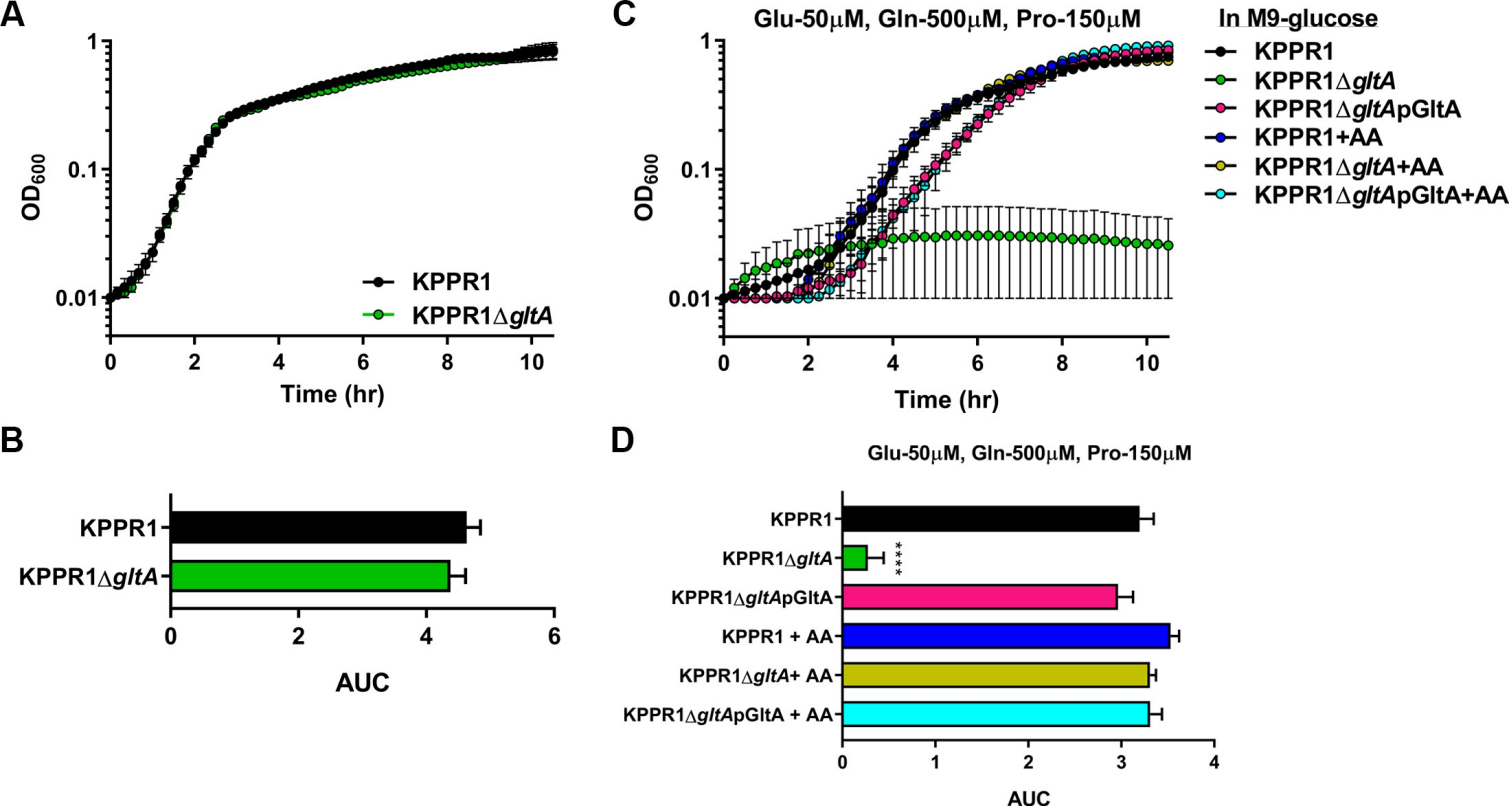
*gltA* is dispensable for growth in mouse serum. (A) WT KPPR1 and KPPR1Δ*gltA* were grown in M9 minimal media + 20% heat-inactivated murine serum without glucose. (B) AUC analysis of WT KPPR1 and KPPR1Δ*gltA* growth in M9 minimal media + 20% heat-inactivated murine serum without glucose (n = 3, Student’s t-test, mean displayed ± SEM). (C) WT KPPR1, KPPR1Δ*gltA*, and KPPR1Δ*gltA*pGltA were grown M9 minimal media + 0.4% glucose with physiological levels of amino acids present in human serum (n = 3, mean displayed ± SEM). (D) AUC analysis of WT KPPR1, KPPR1Δ*gltA*, and KPPR1Δ*gltA*pGltA growth M9 minimal media + 0.4% glucose with physiological levels of amino acids present in human serum (n = 3, ****P < 0.00005 compared to all other groups, Tukey’s multiple comparison test following ANOVA, mean displayed ± SEM). “+AA” label indicates addition of amino acids to growth media at concentrations indicated in graph title.

We next hypothesized that the loss of *gltA* would not affect bacterial growth in a physiologically relevant amino acid rich environment, such as sera [48]. In minimal medium containing 20% heat-inactivated serum, no differences in growth were observed in heat-inactivated serum between WT KPPR1 and KPPR1Δ*gltA* strains (Fig 5A and 5B), and these results were replicated in non-heat-inactivated serum (S7 Fig). To determine if amino acid levels in reported in human blood are sufficient to functionally complement the loss of *gltA*, we tested the growth of WT KPPR1 and KPPR1Δ*gltA* strains in minimal medium with serum-level concentrations of glutamine, glutamate, and proline [49,50], which are higher than those measured in BALF (Table 2). Indeed, serum-level concentrations of glutamine, glutamate, and proline were able to restore growth of the KPPR1Δ*gltA* strain (Fig 5C and 5D). These data support the indication that glutamate family amino acid auxotrophy induced by deletion of *gltA* is the basis of the loss of fitness in amino acid deplete environments, such as the *Lcn2*^*+/+*^ lung.

**Fig 6:**
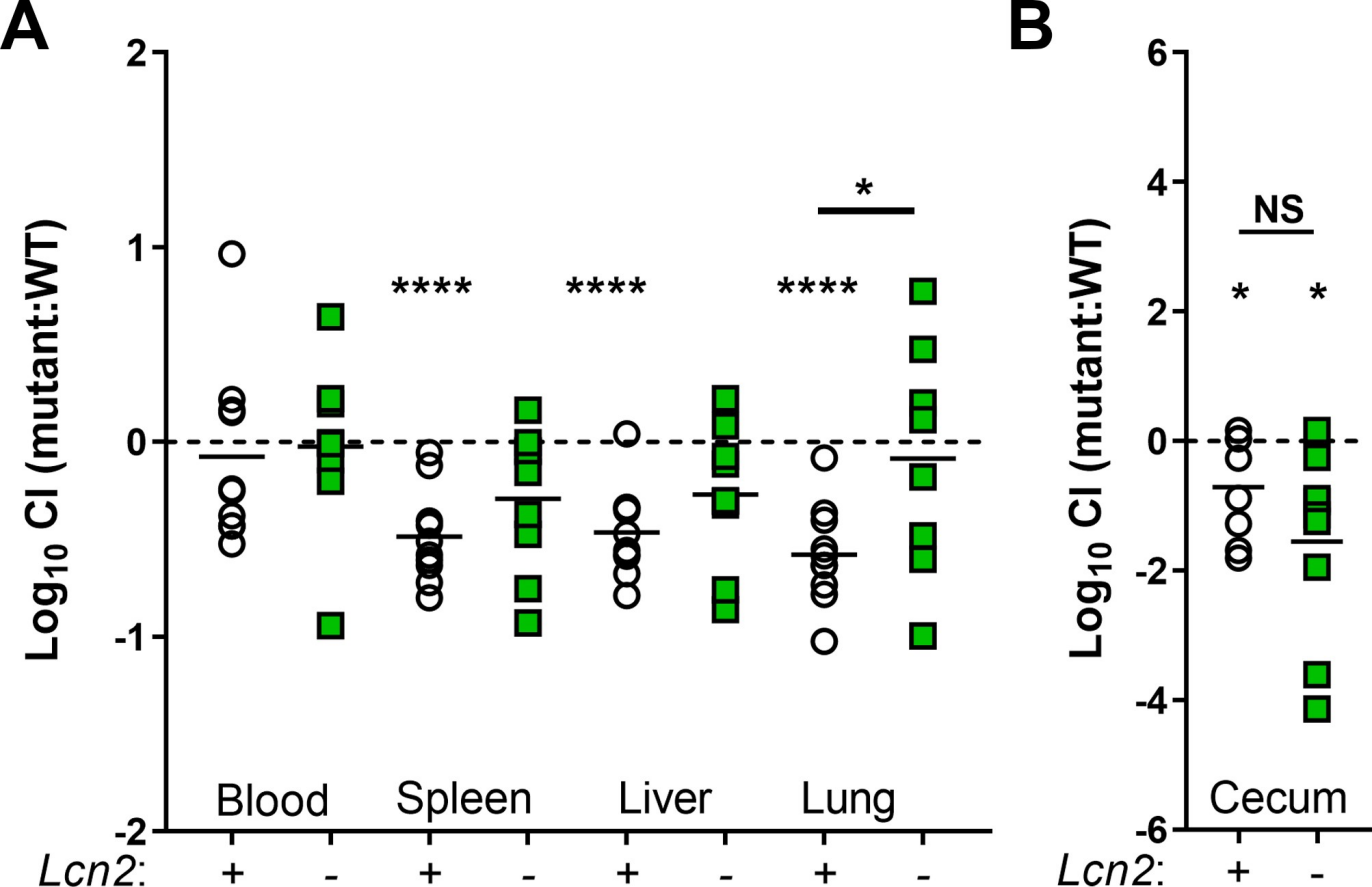
*gltA* influences site-specific fitness during bacteremia and oral infection. (A) C57BL/6J mice or isogenic *Lcn2*^*-/-*^ mice were intraperitoneally inoculated with approximately 5×10^5^ CFU of a 1:1 mix of WT KPPR1 and KPPR1Δ*gltA*. Bacterial burden in the blood, spleen, liver, and lung was measured after 24 hours, and log_10_ competitive index of the mutant strain compared to the WT strain was calculated for each mouse strain (n = 9–11 per group, mean displayed, *P < 0.05, ****P < 0.00005, one-sample *t* test or Student’s *t* test). (B) C57BL/6J mice or isogenic *Lcn2*^*-/-*^ mice were orally inoculated with approximately 5×10^6^ CFU of a 1:1 mix of WT KPPR1 and KPPR1Δ*gltA*. After 48 hours, mice were euthanized, cecum bacterial load was measured, and log_10_ competitive index of the mutant strain compared to the WT strain was calculated for each mouse strain (n = 8–9 per group, mean displayed, *P < 0.05, Student’s *t* test).

The differential necessity of *gltA* for growth in BALF and serum led us to hypothesize that *gltA* may be a fitness factor only in certain body sites where Kp infections occur. To test this hypothesis, we first employed a peritoneal injection murine model of Kp infection. Consistent with results from an aspiration route, KPPR1Δ*gltA* was at a competitive disadvantage compared to WT KPPR1 when reaching the *Lcn2*^*+/+*^ lung through a hematogenous route, and this disadvantage was alleviated in the *Lcn2*^*-/-*^ lung (Fig 6A, S8 Fig). Consistent with *ex vivo* serum growth, KPPR1Δ*gltA* was not competitively disadvantaged in the blood of either mouse background but was less fit in the spleen and liver of *Lcn2*^*+/+*^ mice (Fig 6A, S8 Fig).

Next, we assessed the role of *gltA* in the large intestine. Results from our metabolic screen (Fig 2A, S2 Data) indicate that the KPPR1Δ*gltA* strain is unable to utilize glycolytic substrates without the presence of amino acids that rescue the loss of GltA. Glycolytic metabolism has been shown to be critical for *Escherichia coli* gut colonization [51–53], thus we hypothesized that the KPPR1Δ*gltA* strain may be less fit in the gut. Indeed, the KPPR1Δ*gltA* strain was unable to grow with multiple single sugars, representative of the sugars available during gut colonization as a carbon source (S5 Fig) [51–53]. To test the role of *gltA* in intestinal colonization, we gavaged *Lcn2*^*+/+*^ and *Lcn2*^*-/-*^ mice with approximately 5×10^6^ CFU of a 1:1 mix of WT KPPR1 and KPPR1Δ*gltA*. We found that GltA is a fitness factor for cecal colonization in both the *Lcn2*^*+/+*^ and *Lcn2*^*-/-*^ background (Fig 6B, S8 Fig) and the mutant has a defect in mono-inoculation that approached statistical significance (P = 0.06; S8 Fig).

Finally, we aimed to assess the ability of glutamate family amino acids to alleviate *gltA* deletion-induced auxotrophy in organ homogenates where fitness defects were observed. As was observed with BALF (Fig 4D), the addition of glutamate was sufficient to restore the fitness of the KPPR1Δ*gltA* strain in both spleen and liver organ homogenates from *Lcn2*^*+/+*^ mice (S9 Fig). Proline was sufficient to restore the fitness of the KPPR1Δ*gltA* strain in spleen homogenate (S9 Fig), though not in liver homogenate (S9 Fig). Addition of valine had no impact on the fitness of the KPPR1Δ*gltA* strain; however, complementation of GltA *in trans* was able to partially but significantly alleviate deletion of *gltA* (S9 Fig). Similarly, in cecal homogenates, addition of glutamate completely restored and proline partially restored the fitness of the KPPR1Δ*gltA* strain (S9 Fig), and addition of valine had no impact (S9 Fig). Together, these data show that *gltA* influences site-specific fitness during infection in various body sites through conferring metabolic flexibility, and that *gltA* deletion-induced auxotrophy can be alleviated by the glutamate family of amino acids in these body sites.

## Discussion

The ability of a bacterial cell to utilize available nutrients upon encountering a new environment, referred to as metabolic flexibility, is critical for its survival and success. Bacteria must control highly interconnected metabolic pathways that are quickly activated based on substrate availability in their local environment. Central carbon metabolism connects many pathways in the cell by providing carbon skeletons for biosynthesis of macromolecular building blocks and conversely represents convergence points for the catabolism of macromolecules. Central carbon metabolism is comprised of glycolysis, gluconeogenesis, the Entner-Doudoroff pathway, the pentose phosphate pathway, and the citric acid cycle. The research presented here identifies the citric acid cycle component, citrate (Si)-synthase (GltA), as a critical mediator of metabolic flexibility in Kp, and this metabolic flexibility drastically influences fitness during infection in a site-specific manner. Using multiple murine models of infection in *Lcn2*^*+/+*^ and *Lcn2*^*-/-*^ backgrounds, we show that GltA is a fitness factor during lung infection by direct and hematogenous routes but is not necessary for bacteremia. Additionally, GltA is necessary for gut colonization, which frequently precedes infection [12,13]. The necessity of GltA is likely determined by the nutrient composition of each respective body site, specifically access to amino acids that the bacteria cannot otherwise synthesize *de novo*. This is supported by the observation of differential fitness of KPPR1Δ*gltA* in the *Lcn2*^*+/+*^ and *Lcn2*^*-/-*^ lung, which have different endogenous levels of amino acids. Together, these data provide new insight into how Kp metabolic flexibility determines fitness during infection. While the contributions of specific metabolic processes, such as iron acquisition [24,25,35,36,54–57], nitrogen utilization through urease activity [58], allantoin metabolism [59], and psicose metabolism [60] in Kp pathogenesis have been explored, this study reveals a role for central metabolism and metabolic flexibility during Kp infection.

GltA is a type II citrate synthase, which are characteristically found in Gram-negative bacteria. The Kp GltA, which is ubiquitous in the species, is closely related to the citrate synthases of other members of *Enterobacteriaceae*, such as *Salmonella enterica* and *E*. *coli*, sharing 96% and 95% amino acid sequence identity, respectively. Interestingly, the Kp genome has two genes annotated as *gltA*. Apart from VK055_1802, VK055_2057 is also annotated as *gltA* (hereafter *gltA2*). The *gltA2* gene is universally present in *Klebsiella spp*. but not in other *Enterobacteriaceae* genera. I-TASSER 3D structure prediction [61–63] indicates that GltA2 is structurally similar to GltA despite sharing only 60% and 58% amino acid sequence identity with KPPR1 GltA and *E*. *coli* str. K-12 substr. MG1655 GltA, respectively. Our data demonstrate that GltA and GltA2 are functionally distinct, as the KPPR1Δ*gltA* mutant has a significant defect despite the presence of *gltA2*.

Our data indicate that the loss of *gltA* abrogates the *de novo* synthesis of glutamate and other key carbon skeletons for biosynthesis of amino acids, which is essential to bacterial growth. Glutamate is the fulcrum of glutamine, proline, arginine, and histidine metabolism [64] (S4 Fig). Glutamate is also needed for ammonia assimilation. In fact, glutamate and glutamine provide nitrogen for all nitrogen-containing components of the bacterial cell, and approximately 88% comes from glutamate [65]. Studies in *E*. *coli* have shown that glutamate is the most abundant intracellular metabolite, with an absolute intracellular concentration of 96 mM [66]. Additionally, after conversion to its D-enantiomer, glutamate serves as a component of bacterial peptidoglycan, which forms the cell wall and determines the rate of cell elongation [67]. Thus, Kp lacking *de novo* synthesis of glutamate are incapable of proliferating unless glutamate can be acquired exogenously. This is highlighted by the fact that growth of KPPR1Δ*gltA* in minimal medium is rescued by the addition of glutamate in a dose-dependent manner (S4 Fig). Alternatively, exogenous glutamine, proline, arginine, and histidine can facilitate growth through the central nitrogen metabolic circuit or through production of glutamate through degradation [64]; however, supplementation of α-ketoglutarate at a high concentration was not sufficient for a complete restoration of growth, suggesting a lack of transporting mechanism (Fig 3E and 3F). Additionally, our data demonstrate that dipeptides containing glutamate, glutamine, and histidine can support growth of GltA-deficient Kp (Fig 2C, S2 Data), indicating that Kp could scavenge dipeptides or polypeptides in the course of colonization and infection. Given that the total amino acid and protein concentration was higher in the *Lcn2*^*-/-*^ lung than the *Lcn2*^*+/+*^ lung (Table 2, Fig 4C, S3 Data) it is difficult to identify a single amino acid or polypeptide that rescues the loss of GltA. Rather, it is likely that a combination of free and/or peptide-bound amino acids permits metabolism-dependent niche invasion. Taken together, our findings suggest that stratifying *in vivo* environments as either nutritionally replete or deplete relative to the bacteria is appropriate, and that a systems biology approach of studying bacterial metabolic flexibility is beneficial for understanding the lifestyle of pathogenic bacteria.

By maintaining high metabolic flexibility, pathogenic bacteria can invade multiple niches, and thus, increase their chances of evolutionary success. For example, commensal *E*. *coli* living in the human gut favor glycolytic pathways that take advantage of the sugar-rich mucus lining [51,52], whereas uropathogenic *E*. *coli* (UPEC) favor citric acid cycle-dependent pathways that take advantage of the nitrogen-rich urinary tract environment [68]. As predicted, deletions in the glycolysis, pentose phosphate, and the Entner-Doudoroff pathways have little effect on fitness of *E*. *coli* in the urinary tract environment, whereas specific citric acid cycle components are necessary [9,69]. Similarly, our previous InSeq study indicated the importance of the citric acid cycle during lung infection, identifying *gltA*, *frdA*, and *frdC* as fitness factors during lung infection [26]. Only one glycolytic enzyme (*pfkB*), no pentose phosphate pathway enzymes, and no Entner-Doudoroff pathway enzymes were identified as fitness factors in the previous study [26]. The data presented here support these findings, wherein increased amino acid levels in the *Lcn2*^*-/-*^ lung ameliorated the loss of *gltA*. Interestingly, the only other citric acid cycle enzyme enriched in the *Lcn2*^*-/-*^ lung was the fumarate reductase subunit, *frdD*; however, the other fumarate reductase subunits (*frdA-C*) did not display a similar phenotype (S1 Data), and furthermore, these enzymes are only used during anaerobic growth in *E*. *coli* [70]. Unlike *gltA*, transposon interruptions specific to glycolysis (*pfkB*, VK055_3061 [*pgi*], *tpiA*, *pyk*), the pentose phosphate pathway (*gnd*), and the Entner-Doudoroff pathway (VK055_1337 [*tal1*], VK055_2566 [*tal3*], *edd*) were not alleviated by the presence of increased glutamate family amino acids in the *Lcn2*^*-/-*^ lung (S1 Data), highlighting the importance of *gltA* in conferring metabolic flexibility in this environment. Given that the necessity of *gltA* for complete fitness was seen in some but not all body sites (Fig 6), and loss of *gltA* was alleviated through exogenous supplementation of amino acids (Fig 4D, S9 Fig), our data suggests that metabolic flexibility plays a similar role for Kp as it does for *E*. *coli*, where the ability to utilize available nutrients dictates site-specific fitness. Taken together, our findings indicate that the oxidative citric acid cycle is beneficial for Kp infection by permitting invasion of niches with different nutritional compositions; however, the role of the glycolysis, pentose phosphate pathway, and Entner-Doudoroff pathways during infection of other body sites remains to be explored.

Phenotypic metabolic flexibility has been used to delineate closely related species of the *K*. *pneumoniae* complex, which includes *K*. *pneumoniae*, *K*. *quasipneumoniae*, and *K*. *variicola*, as well as Kp pathogenic lineages. *K*. *quasipneumoniae* is an opportunistic pathogen that is frequently found as a colonizer [71], whereas *K*. *variicola* causes more serious infections [72]. The three members of this complex can be separated by their metabolic profile [73]. Moreover, the Kp strain used in this study, KPPR1, has been shown to be more metabolically flexible than the less pathogenic Kp strain MGH 78578 [74]. Finally, metabolism of D-arabinose [73] and allantoin [59] is associated with hypervirulent Kp strains and a variably-present psicose utilization locus is associated with human infection [60]. As such, metabolic flexibility may be a critical dictator of the variation in clinical outcomes for different *Klebsiella spp*. or Kp pathogenic lineages, and *gltA* is likely a central hub for metabolizing diverse nutrients. Further exploration of the determinants of metabolic flexibility in Kp and the respective association with clinical outcomes is necessary to fully understand how specific metabolic capacity influences fitness during infection.

While this study significantly advances our understanding of the role that metabolic flexibility plays in determining fitness during infection, it is not without limits. Firstly, this study does not address the mechanism underlying the difference in amino acid and protein content between the *Lcn2*^*+/+*^ and *Lcn2*^*-/-*^ lung. In addition to antimicrobial activity, Lcn2 may have the ability to act as a growth and differentiation factor [75,76] and modulate expression of lung epithelial cell genes [25]. Therefore, deletion of *Lcn2* may impact lung homeostasis, leading to the increase in amino acid levels. Although understanding the mechanism underlying this phenotype is beyond the scope of this study, the phenotype served as a useful tool to observe the effect of increased glutamate family amino acid levels on Kp lung fitness. Secondly, this study exclusively uses the Kp strain KPPR1. Additional studies including different strains of the *K*. *pneumoniae* complex are necessary to fully understand the impact of metabolic flexibility during infection. Finally, we were unable to evaluate isocitrate dehydrogenase (*icd)* and the aconitate hydratase (*acnB*) as part of the role of oxidative citric acid cycle in metabolic flexibility during Kp infection. Unfortunately, *icd* and the aconitate hydratase *acnB* were not interrupted in our input pool of transposon mutants (S1 Data). Additionally, the aconitate hydratases *acnA* and *acnB* are able to catalyze the same reaction [77,78], and thus, a growth defect with a single *acnA* mutant may not be expected.

In summary, we have described a novel role for the Kp citrate synthase gene, *gltA*, as a critical mediator of site-specific fitness during infection due to its influence on metabolic flexibility. Taken together, our results represent an advancement in our understanding of Kp metabolism during infection and enhance our knowledge of how these serious infections manifest, such that we are better able to combat these dangerous bacteria.

## Materials and methods

### Ethics statement

This study was performed in strict accordance with the recommendations in the *Guide for the Care and Use of Laboratory Animals* [79]. The University of Michigan Institutional Animal Care and Use Committee approved this research (PRO00007474).

### Materials, media, and bacterial strains

All chemicals were purchased from Sigma-Aldrich (St. Louis, MO) unless otherwise indicated. *K*. *pneumoniae* KPPR1 [27] and isogenic mutants were cultured in Luria-Bertani (LB, Becton, Dickinson and Company, Franklin Lakes, NJ) broth, or in M9 minimal medium (M9 salts [Thermo Fisher Scientific, Waltham, MA], 0.2 M MgSO_4_, 0.01 M CaCl_2_, with or without 0.4% glucose) at 37°C with shaking, or on LB agar at 30°C (Thermo Fisher Scientific). The KPPR1Δ*gltA* mutant was constructed as previously described [26,31]. Briefly, electrocompetent KPPR1 cells containing a modified pKD46 plasmid encoding a spectinomycin resistance cassette [26,31] were electroporated with a *gltA*-specific target site fragment containing a kanamycin resistance cassette isolated from the pKD4 plasmid [31]. Transformants were selected at 37°C on LB agar containing 25 μg/ml kanamycin, re-cultured, and confirmed by colony PCR using flanking primers (S1 Table). The *gltA* complementing plasmid pGltA was constructed using a Gibson Assembly Cloning Kit (New England Biolabs, Ipswich, MA). Briefly, the *gltA* sequence including its promoter was amplified from WT KPPR1 by PCR (S1 Table) and ligated into the pACYC184 backbone [80] to create the pGltA plasmid. The ligation mixture was transformed into NEB 10-beta Competent *E*. *coli* (New England Biolabs) by heat shock. Transformants were selected at 37°C on LB agar containing 30 μg/ml chloramphenicol, re-cultured, and confirmed by colony PCR using (S1 Table). Singe transformants were then grown in batch culture for plasmid extraction using the Plasmid Midi Kit (Qiagen, Germantown, MD). KPPR1Δ*gltA* competent cells were prepared as previously described [26], electroporated with the pGltA plasmid, and selected at 37°C on LB agar containing 30 μg/ml chloramphenicol. Following selection, transformants were re-cultured, and confirmed by colony PCR (S1 Table) and by growth in M9 minimal medium.

### Transposon library construction and InSeq

Construction of the KPPR1 transposon library used in this study has been extensively described elsewhere [26]. Insertion sequencing was performed as previously described [26]. Following infection, total recovered transposon mutants were collected, gDNA was isolated using the DNeasy Blood and Tissue Kit (Qiagen, Germantown, MD), and genomic sequences adjacent to insertion sites were amplified by PCR. Following amplification, Illumina sequencing adapters were ligated to amplified junction DNA fragments, and then fragments were sequenced on an Illumina HiSeq2500 Instrument (Illumina, San Diego, CA). Sequencing reads were filtered, mapped and normalized as described [26].

### Murine models of infection

Six- to 12-week-old C57BL/6J mice (*Lcn2*^*+/+*^, Jackson Laboratory, Jackson, ME) or isogenic *Lcn2*^*-/-*^ mice [28] were used for all murine models of infection. Kp was cultured overnight in LB, then bacteria were pelleted, resuspended, and diluted in sterile phosphate-buffered saline (PBS) to the appropriate dose. For lung infection studies, a dose of 1×10^6^ CFU was used to provide sufficient representation of all mutants in the transposon library [26]. Mice were anesthetized with isoflurane, inoculated in the pharynx, and the mouse was monitored until ambulatory. For validation of InSeq findings, 1×10^6^ CFU was inoculated in the pharynx either as single strains or as a 1:1 mix of the WT and mutant strain. After 24 hours, mice were euthanized by CO_2_ asphyxiation and lungs were collected, weighed, and homogenized in sterile PBS, and homogenates were dilution plated on selective media to determine bacterial load and competitive index. For bacteremia studies, inocula were prepared as above, and mice were inoculated intraperitoneally with approximately 5×10^5^ CFU. After 24 hours, mice were euthanized and bacterial load was assessed as above. For oral inoculation studies, inocula were prepared as above, and mice were inoculated orally with approximately 5×10^6^ CFU. After 48 hours, mice were euthanized and bacterial load was assessed as above. In all models, mice were monitored daily for signs of distress (hunched posture, ruffled fur, decreased mobility, and dehydration) and euthanized at predetermined timepoints. No blinding was performed between experimental groups.

### Preparation of recombinant human lipocalin 2 protein and Lcn2 growth assay

Human lipocalin 2 was recombinantly expressed, purified, and validated as previously described [55,81,82]. WT KPPR1 and various isogenic mutants were grown overnight in LB, then inoculated in RPMI with 10% (v/v) heat-inactivated human serum with or without 1.6 μM purified recombinant human Lcn2 at a concentration of 1 × 10^3^ CFU/mL. Cultures were incubated overnight at 37°C with 5% CO_2_, and bacterial density was enumerated by dilution plating.

### BioLog Phenotype MicroArray analysis

BioLog Phenotype MicroArrays (Biolog, Hayward, CA) analysis was performed in accordance with manufacturer’s instructions with some modifications. WT KPPR1 and KPPR1Δ*gltA* were cultured overnight in LB, then bacteria were pelleted, washed once in sterile PBS, then re-suspended in sterile PBS. Each strain was diluted in IF-0 medium to a final OD_600_ of 0.035, then diluted again to final inoculation concentrations as per manufacturer’s instruction. The final inoculum (100 μL) was plated onto plates PM1, PM2, and PM3. Sodium pyruvate (Thermo Fisher Scientific) was used as a carbon source for PM3 at a final concentration of 2 mM in accordance with previous metabolic phenotype analysis [73]. After inoculation, plates were sealed to avoid cross contamination of volatile compounds produced during Kp growth [73,83] and statically incubated overnight at 37°C. Following overnight incubation, growth was measured at OD_595_.

### Growth curves

The WT KPPR1 and KPPR1Δ*gltA* strains were cultured overnight in LB broth, then diluted to a uniform OD_600_ of 0.01 in the culture medium of interest the following day. Amino acids were supplemented at 10 mM unless otherwise indicated, and carbon sources were supplemented at 5 mg/mL. Cultures were incubated at 37°C with aeration and OD_600_ readings were taken every 15 min using an Eon microplate reader with Gen5 software (Version 2.0, BioTek, Winooski, VT) for up to 24 hours. To simultaneously assess doubling time, growth rate, lag time, non-sigmoidal growth due to stress and bacterial density, area under the curve analysis was used to quantify differences in growth [37,38] using Prism 6 (GraphPad Software, La Jolla, CA).

### Serum growth assay

The WT KPPR1 and KPPR1Δ*gltA* strains were cultured overnight in LB broth with antibiotic supplementation, if necessary. Bacteria were then washed with M9 minimal media by centrifugation, resuspended, and diluted to an OD_600_ of 0.01 in M9 minimal media without glucose, supplemented with 20% (v/v) murine or 10% (v/v) human sera. For some experiments, sera were heat-inactivated at 56°C for 30 minutes. Cultures were grown at 37°C and OD_600_ was measured using an Eon microplate reader with Gen5 software (Version 2.0, BioTek, Winooski, VT) for up to 24 hours.

### BALF and organ homogenate growth assay

Six- to 12-week-old C57BL/6J mice (*Lcn2*^*+/+*^, Jackson Laboratory, Jackson, ME) or isogenic *Lcn2*^*-/-*^ mice [28] were used for BALF and organ collection. Briefly, mice were euthanized by CO_2_ asphyxiation and tracheas were exposed. A small incision was made in the trachea, and polyethylene tubing (external diameter 0.965 mm, internal diameter 0.58 mm, BD, Franklin Lakes, NJ) attached to a 23-gauge luer-stub adaptor and syringe containing 2 mL sterile PBS. Following tubing insertion, 4–0 silk suture (Ethicon, Somerville, NJ) was used to secure the trachea and then the lungs were flushed with PBS. Organs were collected following BALF collection. BALF was kept on ice until processing, wherein BALF was centrifuged at 21,130 x *g* for 30 min at 4°C to pellet contaminating bacteria, then supernatant was stored at -80°C. Rifampin was added to BALF to a final concentration of 30 μg/ml immediately prior to use. Organs were homogenized in 1.5 mL sterile PBS, then sterile filtered through a 0.22 μM PVDF filter (MilliporeSigma, Burlington, MA). The WT KPPR1 and KPPR1Δ*gltA* strains were cultured overnight in LB broth with antibiotic supplementation, if necessary. Bacteria were washed with PBS by centrifugation, resuspended, diluted to an OD_600_ of 0.1 in sterile PBS or sterile PBS with amino acid supplement, then mixed with BALF or organ homogenate from independent mice at a ratio of 1:9 inoculum: BALF/homogenate. Cultures were incubated at 37°C with aeration and OD_600_ was measured using an Eon microplate reader with Gen5 software (Version 2.0, BioTek, Winooski, VT) for up to 24 hours.

### Metabolomic analysis of BALF

For metabolomic analysis 100 μL of BALF was used. Following lipid extraction, BALF protein content was measured by BCA Protein Assay Kit (Thermo Fisher Scientific, Waltham, MA) following manufacturer’s instruction. 20 μL was set aside to create a pooled sample from all study samples, and the remaining 80 μL of each BALF sample was prepared for GC-MS analysis according to manufacturer instructions using the Phenomenex EZFaast Free Amino Acids Analysis GC-MS kit (Phenomenex, Torrance, CA, USA). Briefly, BALF samples were combined with an internal standard (norvaline) and subjected to cation exchange solid phase extraction to purify amino acids from proteins, salts and other matrix components. The amino acids were then derivatized using a proprietary reagent and catalyst, the solvent was evaporated under a gentle nitrogen stream at room temperature, and finally the sample was resuspended for GC analysis. Quality control samples were prepared by pooling equal volumes of each sample and were injected at the beginning and the end of each analysis and after every 10 sample injections to provide a measurement of the system’s stability and performance as well as reproducibility of the sample preparation method. The pooled sample was treated identically to the study samples and was analyzed along with the samples for quality control purposes. Calibration standards were prepared containing all 20 proteinogenic amino acids at concentrations of 10, 25, 50 and 100 μM and were analyzed in replicate along with samples to enable absolute quantitation of amino acids.

GC-MS analysis was performed on an Agilent 69890N GC -5975 MS detector with the following parameters: a 1 μL sample was injected with a 1:15 split ratio on a ZB-AAA 10 m column (Phenomenex, Torrance, CA, USA) with a He gas flow rate of 1.1 mL/min. The GC oven initial temperature was 110°C and was increased at 30°C per minute to 320°C. The inlet temperature was 250°C and the MS-source and quad temperatures were 230° and 150°C respectively. GC-MS data were processed using MassHunter Quantitative Analysis software version B.07.00. Amino acids were quantitated as μM/L BALF using linear calibration curves generated from the standards listed above. To generate these curves, all peak areas in samples and calibration standards were first normalized to the peak area of the internal standard, norvaline. Based on replicate analysis of biological samples, the quantitative variability for all reported amino acids using this method is <15% RSD.

### Statistical analysis

All *in vitro* experimental replicates represent biological replicates. For *in vitro* studies, except metabolomic analysis, two-tailed Student’s *t*-tests or ANOVA followed by Tukey’s multiple comparisons post-hoc test was used to determine significant differences between groups. For metabolomic analysis, R version 3.5 and the “gplots,” “pca3d,” and “rgl” packages were used for data visualization and differences were assessed by two-sample Wilcoxon test and controlled for false discovery rate using the two-stage linear step-up procedure of Benjamini and Hochberg (2006) with Q = 5%. All animal studies except the InSeq study were replicated at least twice. Competitive indices were log transformed and a one-sample *t-*test was used to determine significant differences from a hypothetical value of 0 or two-tailed Student’s *t-*test was used to determine significant differences between groups. All CFU values were log_10_ transformed for analysis. A *P* value of less than 0.05 was considered statistically significant for the above experiments, and analysis was performed using Prism 6 (GraphPad Software, La Jolla, CA). For InSeq analysis, a *P* value was first calculated for each insertion using an exact Poisson test for comparing the two groups, and then the insertion-level *P* values were combined using Fisher's method [84] to obtain the statistical significance for each gene. Finally, the *P* values were adjusted to control the false discovery rate (Dabney A and Storey JD. qvalue: Q-value estimation for false discovery rate control. R package version 1.43.0.). A *P* value of less than 1.3×10^−5^ was considered statistically significant. This led to a list of 1,678 enriched genes, which was then shortened to 49 genes by limiting analysis to genes with a log *Lcn2*^*+/+*^: *Lcn2*^*-/-*^ insertion ratio greater or less than 3 standard deviations from the mean log *Lcn2*^*+/+*^: *Lcn2*^*-/-*^ insertion ratio. 43 of these 49 genes had an FDR adjusted *P* value less than 1.3×10^−5^.

## Supporting information

S1 Fig Summary of comparative InSeq study. (A) C57BL/6J mice or isogenic *Lcn2*^*-/-*^ mice were retropharyngeally inoculated with approximately 1×10^6^ CFU of a pool of ~25,000 transposon mutants to compare mutant frequencies in *Lcn2*^*+/+*^ and *Lcn2*^*-/-*^ mice during lung infection. Twenty-four hours post-inoculation, total lung CFU were collected for DNA extraction, Illumina sequencing of transposon junctions was performed, reads were mapped to the KPPR1 reference genome, and insertion read counts were compared between *Lcn2*^*+/+*^ and *Lcn2*^*-/-*^ mice. (B) The log_10_ insertion count ratio was calculated for each gene. A ratio of 0 indicated that there is no difference in insertion read counts between *Lcn2*^*+/+*^ and *Lcn2*^*-/-*^ mice, thus indicating that the KPPR1 gene does not have any interaction with Lcn2. Genes with log_10_ insertion read count ratios greater than or less than 3 standard deviations from the mean (mean ± S.D. = 0.094 ± 0.53) suggest a strong interaction between that gene and Lcn2. 49 genes meet this criterion. (C) Volcano plot summarizing the calculated *P* values of each log_10_ insertion read count difference counts between *Lcn2*^*+/+*^ and *Lcn2*^*-/-*^ mice. The dotted line (significant *P* value cutoff = 1.3×10^−5^) indicates the threshold for consideration of a *P* value as significant after correction for multiple comparisons. 43 of 49 genes with log_10_ insertion read count ratios greater than or less than 3 standard deviations from the mean have a *P* value < 1.3×10^−5^, including *gltA*, which is shown in black. (D) Locations and frequencies of transposon insertions in *gltA*. (E) WT KPPR1, KPPR1Δ*gltA*, and KPPR1Δ*gltA*pGltA were grown in LB (n = 3, mean displayed ± SEM). (F) Area under curve analysis of WT KPPR1, KPPR1Δ*gltA*, and KPPR1Δ*gltA*pGltA growth in LB (n = 3, Tukey’s multiple comparison test following ANOVA, mean displayed ± SEM).
(TIF)

S2 Fig Summary of bacterial burden during lung infection. (A) C57BL/6J mice or isogenic *Lcn2*^*-/-*^ mice were retropharyngeally inoculated with approximately 1×10^6^ CFU of a 1:1 mix of WT KPPR1 and KPPR1Δ*gltA* and lung bacterial burden was measured after 24 hours (n = 10 per group, mean displayed, *P < 0.05, ***P < 0.0005, Student’s *t* test). (B) C57BL/6J mice were retropharyngeally inoculated with approximately 1×10^6^ CFU of either WT KPPR1 or KPPR1Δ*gltA* and lung bacterial burden was measured after 24 hours (n = 10 per group, mean displayed, *P < 0.05, Student’s *t* test).
(TIF)

S3 Fig Loss of *gltA* is not complemented by citrate the in the presence of a glycolytic substrate, but is complemented by citrate and glutamate when provided as a gluconeogenic substrate. WT KPPR1 and KPPR1Δ*gltA* were grown in M9 minimal media with (A) 10 mM citrate, (B) 0.4% glucose, or (C) 0.4% glucose and 10 mM citrate (n = 3, mean displayed ± SEM). (D) AUC analysis of WT KPPR1 and KPPR1Δ*gltA* grown in M9 minimal media with 10 mM citrate, 0.4% glucose, or 0.4% glucose and 10 mM citrate (n = 3, ***P < 0.0005, ****P < 0.00005, Student’s *t* test, mean displayed ± SEM).
(TIF)

S4 Fig Summary of the roles of glutamate family amino acids. Glutamate family amino acids enter the citric acid cycle via α-ketoglutarate and exit the citric acid cycle for protein and peptidoglycan biosynthesis or nitrogen assimilation after conversion to glutamate. Glutamate plays a central role in these processes due to its position as a hub of multiple metabolic pathways. Deletion of *gltA* inhibits the ability of Kp to use metabolic substrates outside of the glutamate family of amino acids, resulting in significantly less metabolic flexibility.
(TIF)

S5 Fig GltA is necessary for glycolytic substrate utilization. WT KPPR1 and KPPR1Δ*gltA* were grown in M9 minimal media + 5 mg/mL of (A) acetate, (B) arabinose, (C) fucose, (D) galactose, (E), gluconate, (F) glucose, (G) lactose, (H) pyruvate, (I) raffinose, (J) rhamnose, or (K) xylose (n = 3, mean displayed ± SEM). (L) AUC analysis of WT KPPR1 and KPPR1Δ*gltA* growth in M9 minimal media + 5 mg/mL specific sugar (n = 3, **P < 0.005, ***P < 0.005, Student’s t-test, mean displayed ± SEM).
(TIF)

S6 Fig Auxotrophy due to deletion of *gltA* is rescued by glutamate in a dose-dependent manner. (A) WT KPPR1 and KPPR1Δ*gltA* were grown in M9 minimal media + 0.4% glucose with increasing concentrations of glutamate (n = 3, mean displayed ± SEM). (B) AUC analysis of WT KPPR1 and KPPR1Δ*gltA* were grown in M9 minimal media + 0.4% glucose with increasing concentrations of glutamate (n = 3, ****P < 0.00005, Tukey’s multiple comparison test following ANOVA, mean displayed ± SEM).
(TIF)

S7 Fig *gltA* growth in non-heat-inactivated murine serum. (A) WT KPPR1 and KPPR1Δ*gltA* were grown in M9 minimal media + 20% non-heat-inactivated murine serum (n = 3, mean displayed ± SEM). (B) AUC analysis of WT KPPR1 and KPPR1Δ*gltA* growth in M9 minimal media + 20% non-heat-inactivated murine serum (n = 3, **P < 0.005, Student’s t-test, mean displayed ± SEM).
(TIF)

S8 Fig Summary of bacterial burden during bacteremia and oral infection. (A) C57BL/6J mice or isogenic *Lcn2*^*-/-*^ mice were intraperitoneally inoculated with approximately 1×10^6^ CFU of a 1:1 mix of WT KPPR1 and KPPR1Δ*gltA* and bacterial burden was measured after 24 hours (n = 9–11 per group, mean displayed, *P < 0.05, Student’s *t* test). (B) C57BL/6J mice or isogenic *Lcn2*^*-/-*^ mice were orally inoculated with approximately 5×10^6^ CFU of a 1:1 mix of WT KPPR1 or KPPR1Δ*gltA* and cecal bacterial burden was measured after 48 hours (n = 8–9 per group, mean displayed, Mann-Whitney test). (C) C57BL/6J mice were orally inoculated with approximately 1×10^6^ CFU of WT KPPR1 or KPPR1Δ*gltA* and cecal bacterial burden was measured after 48 hours (n = 9–10 per group, mean displayed, Mann-Whitney test).
(TIF)

S9 Fig Loss of GltA is rescued by glutamate family amino acids in *Lcn2^+/+^* organ homogenates. Murine (A) spleen, (B) liver, and (C) cecum homogenate generated from uninfected C57BL/6J mice with or without amino acids was inoculated with a 1:1 mix of WT KPPR1 and KPPR1Δ*gltA* or WT KPPR1 and KPPR1Δ*gltA*pGltA. Bacterial burden was measured after 24 hours, and log_10_ competitive index of the mutant strain compared to the WT strain was calculated for each sample (n = 4 mice per group, *P < 0.05, **P < 0.005, ***P < 0.0005, ****P < 0.00005, one-sample *t* test or Tukey’s multiple comparison test following ANOVA).
(TIF)

S1 Data Summary of comparative InSeq data. (XLSX)

S2 Data Summary of BioLog Phenotype MicroArray data. (XLSX)

S3 Data Summary of individual amino acid and protein concentrations from *Lcn2^+/+^* and *Lcn2^-/-^* BALF. (XLSX)

S1 Table Primers used in this study. (XLSX)

## References

[1] Navon-Venezia S, Kondratyeva K, Carattoli A (2017) *Klebsiella pneumoniae*: a major worldwide source and shuttle for antibiotic resistance. FEMS Microbiol Rev 41: 252–275. 10.1093/femsre/fux013 28521338

[2] Castanheira M, Farrell SE, Krause KM, Jones RN, Sader HS (2014) Contemporary diversity of beta-lactamases among Enterobacteriaceae in the nine U.S. census regions and ceftazidime-avibactam activity tested against isolates producing the most prevalent beta-lactamase groups. Antimicrob Agents Chemother 58: 833–838. 10.1128/AAC.01896-13 24247134 PMC3910895

[3] Magill SS, Edwards JR, Bamberg W, Beldavs ZG, Dumyati G, et al (2014) Multistate Point-Prevalence Survey of Health Care–Associated Infections. New England Journal of Medicine 370: 1198–1208. 10.1056/NEJMoa1306801 24670166 PMC4648343

[4] Munoz-Price LS, Poirel L, Bonomo RA, Schwaber MJ, Daikos GL, et al (2013) Clinical epidemiology of the global expansion of *Klebsiella pneumoniae* carbapenemases. Lancet Infect Dis 13: 785–796. 10.1016/S1473-3099(13)70190-7 23969216 PMC4673667

[5] Lee CR, Lee JH, Park KS, Jeon JH, Kim YB, et al (2017) Antimicrobial Resistance of Hypervirulent *Klebsiella pneumoniae*: Epidemiology, Hypervirulence-Associated Determinants, and Resistance Mechanisms. Front Cell Infect Microbiol 7: 483 10.3389/fcimb.2017.00483 29209595 PMC5702448

[6] Bialek-Davenet S, Criscuolo A, Ailloud F, Passet V, Jones L, et al (2014) Genomic definition of hypervirulent and multidrug-resistant *Klebsiella pneumoniae* clonal groups. Emerg Infect Dis 20: 1812–1820. 10.3201/eid2011.140206 25341126 PMC4214299

[7] Rohmer L, Hocquet D, Miller SI (2011) Are pathogenic bacteria just looking for food? Metabolism and microbial pathogenesis. Trends Microbiol 19: 341–348. 10.1016/j.tim.2011.04.003 21600774 PMC3130110

[8] Radlinski LC, Brunton J, Steele S, Taft-Benz S, Kawula TH (2018) Defining the Metabolic Pathways and Host-Derived Carbon Substrates Required for *Francisella tularensis* Intracellular Growth. MBio 9. 10.1128/mBio.01471-18 PMC6247087 30459188

[9] Alteri CJ, Himpsl SD, Mobley HL (2015) Preferential use of central metabolism in vivo reveals a nutritional basis for polymicrobial infection. PLoS Pathog 11: e1004601 10.1371/journal.ppat.1004601 25568946 PMC4287612

[10] Alteri CJ, Mobley HL (2012) *Escherichia coli* physiology and metabolism dictates adaptation to diverse host microenvironments. Curr Opin Microbiol 15: 3–9. 10.1016/j.mib.2011.12.004 22204808 PMC3265668

[11] Winter SE, Thiennimitr P, Winter MG, Butler BP, Huseby DL, et al (2010) Gut inflammation provides a respiratory electron acceptor for Salmonella. Nature 467: 426–429. 10.1038/nature09415 20864996 PMC2946174

[12] Martin RM, Bachman MA (2018) Colonization, Infection, and the Accessory Genome of *Klebsiella pneumoniae*. Front Cell Infect Microbiol 8: 4 10.3389/fcimb.2018.00004 29404282 PMC5786545

[13] Martin RM, Cao J, Brisse S, Passet V, Wu W, et al (2016) Molecular Epidemiology of Colonizing and Infecting Isolates of *Klebsiella pneumoniae*. mSphere 1(5):e00261–16 10.1128/mSphere.00261-16 27777984 PMC5071533

[14] Gorrie CL, Mirceta M, Wick RR, Edwards DJ, Thomson NR, et al (2017) Gastrointestinal Carriage Is a Major Reservoir of *Klebsiella pneumoniae* Infection in Intensive Care Patients. Clin Infect Dis 65: 208–215. 10.1093/cid/cix270 28369261 PMC5850561

[15] Johanson WG Jr., Pierce AK, Sanford JP, Thomas GD (1972) Nosocomial respiratory infections with gram-negative bacilli. The significance of colonization of the respiratory tract. Ann Intern Med 77: 701–706. 10.7326/0003-4819-77-5-701 5081492

[16] Rosenthal S, Tager IB (1975) Prevalence of gram-negative rods in the normal pharyngeal flora. Ann Intern Med 83: 355–357. 10.7326/0003-4819-83-3-355 810051

[17] Thom BT (1970) Klebsiella in faeces. Lancet 2: 1033. 10.1016/s0140-6736(70)92845-x 4098067

[18] Weinberg ED (1975) Nutritional immunity. Host's attempt to withold iron from microbial invaders. Jama 231: 39–41. 10.1001/jama.231.1.39 1243565

[19] Baumler AJ, Sperandio V (2016) Interactions between the microbiota and pathogenic bacteria in the gut. Nature 535: 85–93. 10.1038/nature18849 27383983 PMC5114849

[20] Hood MI, Skaar EP (2012) Nutritional immunity: transition metals at the pathogen-host interface. Nat Rev Microbiol 10: 525–537. 10.1038/nrmicro2836 22796883 PMC3875331

[21] Kehl-Fie TE, Skaar EP (2010) Nutritional immunity beyond iron: a role for manganese and zinc. Curr Opin Chem Biol 14: 218–224. 10.1016/j.cbpa.2009.11.008 20015678 PMC2847644

[22] Holden VI, Bachman MA (2015) Diverging roles of bacterial siderophores during infection. Metallomics 7: 986–995. 10.1039/c4mt00333k 25745886

[23] Goetz DH, Holmes MA, Borregaard N, Bluhm ME, Raymond KN, et al (2002) The neutrophil lipocalin NGAL is a bacteriostatic agent that interferes with siderophore-mediated iron acquisition. Mol Cell 10: 1033–1043. 12453412 10.1016/s1097-2765(02)00708-6

[24] Bachman MA, Oyler JE, Burns SH, Caza M, Lepine F, et al (2011) *Klebsiella pneumoniae* yersiniabactin promotes respiratory tract infection through evasion of lipocalin 2. Infect Immun 79: 3309–3316. 10.1128/IAI.05114-11 21576334 PMC3147564

[25] Holden VI, Lenio S, Kuick R, Ramakrishnan SK, Shah YM, et al (2014) Bacterial siderophores that evade or overwhelm lipocalin 2 induce hypoxia inducible factor 1alpha and proinflammatory cytokine secretion in cultured respiratory epithelial cells. Infect Immun 82: 3826–3836. 10.1128/IAI.01849-14 24980968 PMC4187820

[26] Bachman MA, Breen P, Deornellas V, Mu Q, Zhao L, et al (2015) Genome-Wide Identification of *Klebsiella pneumoniae* Fitness Genes during Lung Infection. MBio 6: e00775 10.1128/mBio.00775-15 26060277 PMC4462621

[27] Broberg CA, Wu W, Cavalcoli JD, Miller VL, Bachman MA (2014) Complete Genome Sequence of *Klebsiella pneumoniae* Strain ATCC 43816 KPPR1, a Rifampin-Resistant Mutant Commonly Used in Animal, Genetic, and Molecular Biology Studies. Genome Announc 2. 10.1128/genomeA.00924-14 PMC4175196 25291761

[28] Flo TH, Smith KD, Sato S, Rodriguez DJ, Holmes MA, et al (2004) Lipocalin 2 mediates an innate immune response to bacterial infection by sequestrating iron. Nature 432: 917–921. 10.1038/nature03104 15531878

[29] Kanehisa M, Sato Y, Kawashima M, Furumichi M, Tanabe M (2016) KEGG as a reference resource for gene and protein annotation. Nucleic Acids Res 44: D457–462. 10.1093/nar/gkv1070 26476454 PMC4702792

[30] Bloxham DP, Herbert CJ, Ner SS, Drabble WT (1983) Citrate synthase activity in *Escherichia coli* harbouring hybrid plasmids containing the *gltA* gene. J Gen Microbiol 129: 1889–1897. 10.1099/00221287-129-6-1889 6355385

[31] Datsenko KA, Wanner BL (2000) One-step inactivation of chromosomal genes in *Escherichia coli* K-12 using PCR products. Proc Natl Acad Sci U S A 97: 6640–6645. 10.1073/pnas.120163297 10829079 PMC18686

[32] Guerinot ML, Meidl EJ, Plessner O (1990) Citrate as a siderophore in *Bradyrhizobium japonicum*. J Bacteriol 172: 3298–3303. 10.1128/jb.172.6.3298-3303.1990 2140566 PMC209139

[33] Konetschny-Rapp S, Jung G, Meiwes J, Zahner H (1990) Staphyloferrin A: a structurally new siderophore from staphylococci. Eur J Biochem 191: 65–74. 10.1111/j.1432-1033.1990.tb19094.x 2379505

[34] Gross R, Engelbrecht F, Braun V (1985) Identification of the genes and their polypeptide products responsible for aerobactin synthesis by pColV plasmids. Mol Gen Genet 201: 204–212. 10.1007/bf00425661 3003525

[35] Bachman MA, Lenio S, Schmidt L, Oyler JE, Weiser JN (2012) Interaction of lipocalin 2, transferrin, and siderophores determines the replicative niche of *Klebsiella pneumoniae* during pneumonia. MBio 3. 10.1128/mBio.00224-11 PMC3509427 23169997

[36] Holden VI, Wright MS, Houle S, Collingwood A, Dozois CM, et al (2018) Iron Acquisition and Siderophore Release by Carbapenem-Resistant Sequence Type 258 *Klebsiella pneumoniae*. mSphere 3. 10.1128/mSphere.00125-18 PMC5907654 29669884

[37] Tonner PD, Darnell CL, Engelhardt BE, Schmid AK (2017) Detecting differential growth of microbial populations with Gaussian process regression. Genome Res 27: 320–333. 10.1101/gr.210286.116 27864351 PMC5287237

[38] Todor H, Dulmage K, Gillum N, Bain JR, Muehlbauer MJ, et al (2014) A transcription factor links growth rate and metabolism in the hypersaline adapted archaeon *Halobacterium salinarum*. Mol Microbiol 93: 1172–1182. 10.1111/mmi.12726 25060603

[39] Berg J, Tymoczko J, Stryer L (2002) Biochemistry 5th Edition Section 16.2, The Glycolytic Pathway Is Tightly Controlled. New York: W H Freeman.

[40] Bochner BR (1989) Sleuthing out bacterial identities. Nature 339: 157–158. 10.1038/339157a0 2654644

[41] Brosnan JT (2000) Glutamate, at the interface between amino acid and carbohydrate metabolism. J Nutr 130: 988s–990s. 10.1093/jn/130.4.988S 10736367

[42] van Heeswijk WC, Westerhoff HV, Boogerd FC (2013) Nitrogen assimilation in *Escherichia coli*: putting molecular data into a systems perspective. Microbiol Mol Biol Rev 77: 628–695. 10.1128/MMBR.00025-13 24296575 PMC3973380

[43] Dougherty TJ, Thanassi JA, Pucci MJ (1993) The *Escherichia coli* mutant requiring D-glutamic acid is the result of mutations in two distinct genetic loci. J Bacteriol 175: 111–116. 10.1128/jb.175.1.111-116.1993 8093236 PMC196103

[44] Hong JH, Lee WC, Hsu YM, Liang HJ, Wan CH, et al (2014) Characterization of the biochemical effects of naphthalene on the mouse respiratory system using NMR-based metabolomics. J Appl Toxicol 34: 1379–1388. 10.1002/jat.2970 24478122

[45] Grahl N, Puttikamonkul S, Macdonald JM, Gamcsik MP, Ngo LY, et al (2011) In vivo hypoxia and a fungal alcohol dehydrogenase influence the pathogenesis of invasive pulmonary aspergillosis. PLoS Pathog 7: e1002145 10.1371/journal.ppat.1002145 21811407 PMC3141044

[46] Hu JZ, Rommereim DN, Minard KR, Woodstock A, Harrer BJ, et al (2008) Metabolomics in lung inflammation:a high-resolution (1)h NMR study of mice exposedto silica dust. Toxicol Mech Methods 18: 385–398. 10.1080/15376510701611032 20020862 PMC2890313

[47] Evans CR, Karnovsky A, Kovach MA, Standiford TJ, Burant CF, et al (2014) Untargeted LC-MS metabolomics of bronchoalveolar lavage fluid differentiates acute respiratory distress syndrome from health. J Proteome Res 13: 640–649. 10.1021/pr4007624 24289193 PMC4068805

[48] Stein WH, Moore S (1954) The free amino acids of human blood plasma. J Biol Chem 211: 915–926. 13221597

[49] Hisamatsu T, Okamoto S, Hashimoto M, Muramatsu T, Andou A, et al (2012) Novel, objective, multivariate biomarkers composed of plasma amino acid profiles for the diagnosis and assessment of inflammatory bowel disease. PLoS One 7: e31131 10.1371/journal.pone.0031131 22303484 PMC3269436

[50] Smolenska Z, Smolenski RT, Zdrojewski Z (2016) Plasma concentrations of amino acid and nicotinamide metabolites in rheumatoid arthritis—potential biomarkers of disease activity and drug treatment. Biomarkers 21: 218–224. 10.3109/1354750X.2015.1130746 26811910

[51] Chang DE, Smalley DJ, Tucker DL, Leatham MP, Norris WE, et al (2004) Carbon nutrition of Escherichia coli in the mouse intestine. Proc Natl Acad Sci U S A 101: 7427–7432. 10.1073/pnas.0307888101 15123798 PMC409935

[52] Fabich AJ, Jones SA, Chowdhury FZ, Cernosek A, Anderson A, et al (2008) Comparison of carbon nutrition for pathogenic and commensal *Escherichia coli* strains in the mouse intestine. Infect Immun 76: 1143–1152. 10.1128/IAI.01386-07 18180286 PMC2258830

[53] Conway T, Cohen PS (2015) Commensal and Pathogenic *Escherichia coli* Metabolism in the Gut. Microbiol Spectr 3. 10.1128/microbiolspec.MBP-0006-2014 PMC4510460 26185077

[54] Holden VI, Breen P, Houle S, Dozois CM, Bachman MA (2016) *Klebsiella pneumoniae* Siderophores Induce Inflammation, Bacterial Dissemination, and HIF-1alpha Stabilization during Pneumonia. mBio 7. 10.1128/mBio.01397-16 PMC5021805 27624128

[55] Bachman MA, Miller VL, Weiser JN (2009) Mucosal lipocalin 2 has pro-inflammatory and iron-sequestering effects in response to bacterial enterobactin. PLoS Pathog 5: e1000622 10.1371/journal.ppat.1000622 19834550 PMC2757716

[56] Lawlor MS, O'Connor C, Miller VL (2007) Yersiniabactin is a virulence factor for *Klebsiella pneumoniae* during pulmonary infection. Infect Immun 75: 1463–1472. 10.1128/IAI.00372-06 17220312 PMC1828572

[57] Hsieh PF, Lin TL, Lee CZ, Tsai SF, Wang JT (2008) Serum-induced iron-acquisition systems and TonB contribute to virulence in *Klebsiella pneumoniae* causing primary pyogenic liver abscess. J Infect Dis 197: 1717–1727. 10.1086/588383 18433330

[58] Maroncle N, Rich C, Forestier C (2006) The role of *Klebsiella pneumoniae* urease in intestinal colonization and resistance to gastrointestinal stress. Res Microbiol 157: 184–193. 10.1016/j.resmic.2005.06.006 16139482

[59] Chou HC, Lee CZ, Ma LC, Fang CT, Chang SC, et al (2004) Isolation of a chromosomal region of *Klebsiella pneumoniae* associated with allantoin metabolism and liver infection. Infect Immun 72: 3783–3792. 10.1128/IAI.72.7.3783-3792.2004 15213119 PMC427404

[60] Martin RM, Cao J, Wu W, Zhao L, Manthei DM, et al (2018) Identification of Pathogenicity-Associated Loci in Klebsiella pneumoniae from Hospitalized Patients. mSystems 3. 10.1128/mSystems.00015-18 PMC6020474 29963640

[61] Yang J, Yan R, Roy A, Xu D, Poisson J, et al (2015) The I-TASSER Suite: protein structure and function prediction. Nat Methods 12: 7–8. 10.1038/nmeth.3213 25549265 PMC4428668

[62] Roy A, Kucukural A, Zhang Y (2010) I-TASSER: a unified platform for automated protein structure and function prediction. Nat Protoc 5: 725–738. 10.1038/nprot.2010.5 20360767 PMC2849174

[63] Zhang Y (2008) I-TASSER server for protein 3D structure prediction. BMC Bioinformatics 9: 40 10.1186/1471-2105-9-40 18215316 PMC2245901

[64] Neidhardt FC, Curtiss R (1996) *Escherichia coli* and Salmonella: cellular and molecular biology. Washington, D.C.: ASM Press.

[65] Wohlheuter R, Schutt H, Holzer H (1973) The Enzymes of Glutamine Metabolism. Academic Press, New York pp. 45–64.

[66] Bennett BD, Kimball EH, Gao M, Osterhout R, Van Dien SJ, et al (2009) Absolute metabolite concentrations and implied enzyme active site occupancy in *Escherichia coli*. Nat Chem Biol 5: 593–599. 10.1038/nchembio.186 19561621 PMC2754216

[67] Rojas E, Theriot JA, Huang KC (2014) Response of *Escherichia coli* growth rate to osmotic shock. Proc Natl Acad Sci U S A 111: 7807–7812. 10.1073/pnas.1402591111 24821776 PMC4040581

[68] Roesch PL, Redford P, Batchelet S, Moritz RL, Pellett S, et al (2003) Uropathogenic *Escherichia coli* use d-serine deaminase to modulate infection of the murine urinary tract. Mol Microbiol 49: 55–67. 10.1046/j.1365-2958.2003.03543.x 12823810

[69] Alteri CJ, Smith SN, Mobley HL (2009) Fitness of *Escherichia coli* during urinary tract infection requires gluconeogenesis and the TCA cycle. PLoS Pathog 5: e1000448 10.1371/journal.ppat.1000448 19478872 PMC2680622

[70] Jones HM, Gunsalus RP (1987) Regulation of *Escherichia coli* fumarate reductase (frdABCD) operon expression by respiratory electron acceptors and the fnr gene product. J Bacteriol 169: 3340–3349. 10.1128/jb.169.7.3340-3349.1987 3298218 PMC212388

[71] Holt KE, Wertheim H, Zadoks RN, Baker S, Whitehouse CA, et al (2015) Genomic analysis of diversity, population structure, virulence, and antimicrobial resistance in *Klebsiella pneumoniae*, an urgent threat to public health. Proc Natl Acad Sci U S A 112: E3574–3581. 10.1073/pnas.1501049112 26100894 PMC4500264

[72] Maatallah M, Vading M, Kabir MH, Bakhrouf A, Kalin M, et al (2014) *Klebsiella variicola* is a frequent cause of bloodstream infection in the stockholm area, and associated with higher mortality compared to K. pneumoniae. PLoS One 9: e113539 10.1371/journal.pone.0113539 25426853 PMC4245126

[73] Blin C, Passet V, Touchon M, Rocha EPC, Brisse S (2017) Metabolic diversity of the emerging pathogenic lineages of *Klebsiella pneumoniae*. Environ Microbiol 19: 1881–1898. 10.1111/1462-2920.13689 28181409

[74] Henry CS, Rotman E, Lathem WW, Tyo KE, Hauser AR, et al (2017) Generation and Validation of the iKp1289 Metabolic Model for *Klebsiella pneumoniae* KPPR1. J Infect Dis 215: S37–s43. 10.1093/infdis/jiw465 28375518 PMC5790149

[75] Schmidt-Ott KM, Mori K, Li JY, Kalandadze A, Cohen DJ, et al (2007) Dual action of neutrophil gelatinase-associated lipocalin. J Am Soc Nephrol 18: 407–413. 10.1681/ASN.2006080882 17229907

[76] Wang Y, Jia M, Yan X, Cao L, Barnes PJ, et al (2017) Increased neutrophil gelatinase-associated lipocalin (NGAL) promotes airway remodelling in chronic obstructive pulmonary disease. Clin Sci (Lond) 131: 1147–1159. 28381600 10.1042/CS20170096

[77] Gruer MJ, Guest JR (1994) Two genetically-distinct and differentially-regulated aconitases (AcnA and AcnB) in *Escherichia coli*. Microbiology 140 (Pt 10): 2531–2541. 8000525 10.1099/00221287-140-10-2531

[78] Jordan PA, Tang Y, Bradbury AJ, Thomson AJ, Guest JR (1999) Biochemical and spectroscopic characterization of *Escherichia coli* aconitases (AcnA and AcnB). Biochem J 344 Pt 3: 739–746. 10585860 PMC1220695

[79] (2011) Guide for the Care and Use of Laboratory Animals: Eighth Edition; Council NR, editor. Washington, DC: The National Academies Press 246 p. 10.1258/la.2010.010031

[80] Chang AC, Cohen SN (1978) Construction and characterization of amplifiable multicopy DNA cloning vehicles derived from the P15A cryptic miniplasmid. J Bacteriol 134: 1141–1156. 149110 10.1128/jb.134.3.1141-1156.1978 PMC222365

[81] Yang J, Goetz D, Li JY, Wang W, Mori K, et al (2002) An iron delivery pathway mediated by a lipocalin. Mol Cell 10: 1045–1056. 12453413 10.1016/s1097-2765(02)00710-4

[82] Bundgaard JR, Sengelov H, Borregaard N, Kjeldsen L (1994) Molecular cloning and expression of a cDNA encoding NGAL: a lipocalin expressed in human neutrophils. Biochem Biophys Res Commun 202: 1468–1475. 10.1006/bbrc.1994.2096 8060329

[83] Bos LD, Sterk PJ, Schultz MJ (2013) Volatile metabolites of pathogens: a systematic review. PLoS Pathog 9: e1003311 10.1371/journal.ppat.1003311 23675295 PMC3649982

[84] Fisher RA (1992) Statistical methods for research workers Breakthroughs in statistics: Springer pp. 66–70.

