# Supplementary figures and images for: The Klebsiella pneumoniae citrate synthase gene, gltA, influences site specific fitness during infection
Source: PLoS Pathog. 2019 Aug 26;15(8):e1008010. doi: 10.1371/journal.ppat.1008010 (PMC6730947; doi:10.1371/journal.ppat.1008010)

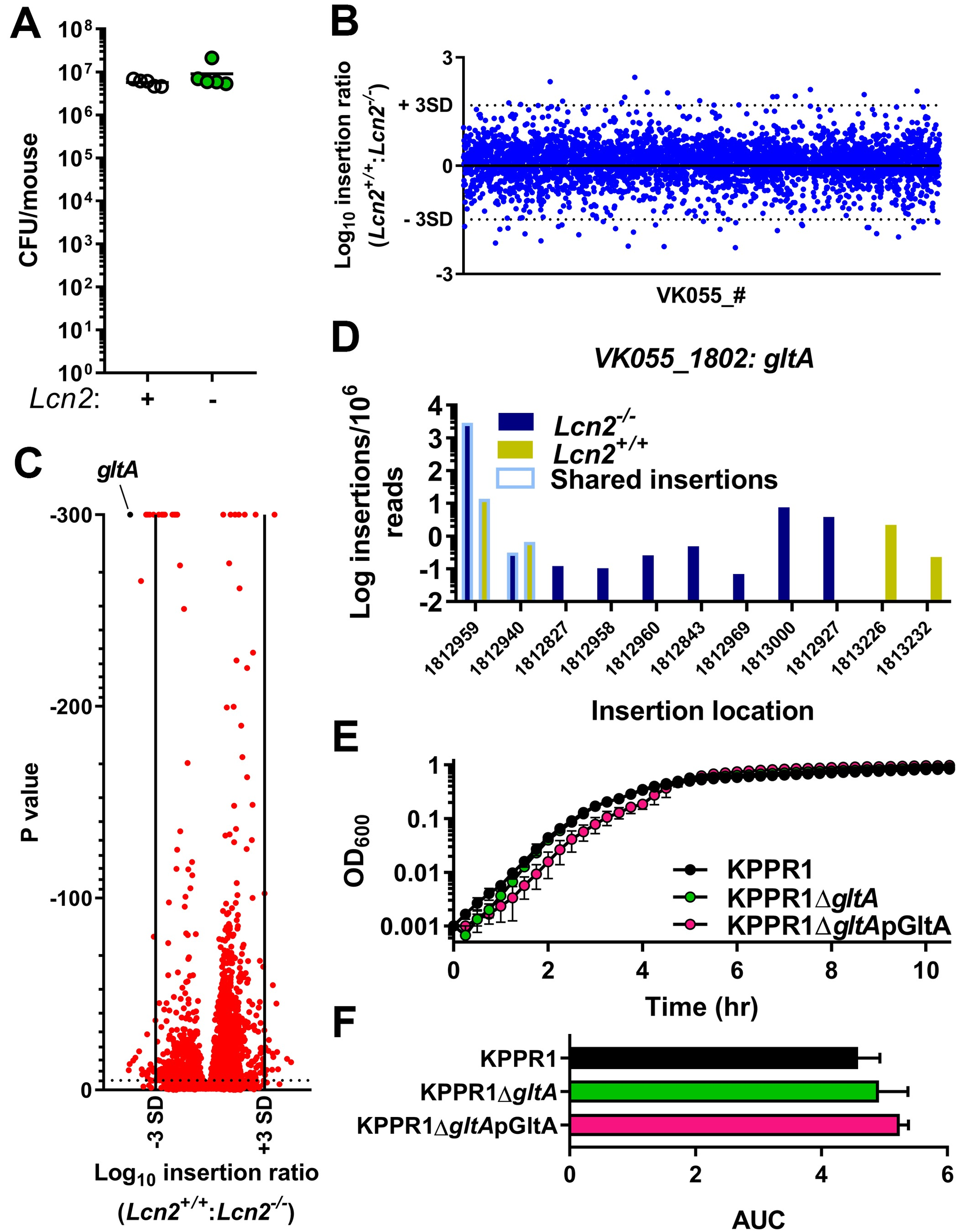

Supplement: S1 Fig — (A) C57BL/6J mice or isogenic Lcn2-/- mice were retropharyngeally inoculated with approximately 1×10^6^ CFU of a pool of ~25,000 transposon mutants to compare mutant frequencies in Lcn2+/+ and Lcn2-/- mice during lung infection. Twenty-four hours post-inoculation, total lung CFU were collected for DNA extraction, Illumina sequencing of transposon junctions was performed, reads were mapped to the KPPR1 reference genome, and insertion read counts were compared between Lcn2+/+ and Lcn2-/- mice. (B) The log10 insertion count ratio was calculated for each gene. A ratio of 0 indicated that there is no difference in insertion read counts between Lcn2+/+ and Lcn2-/- mice, thus indicating that the KPPR1 gene does not have any interaction with Lcn2. Genes with log10 insertion read count ratios greater than or less than 3 standard deviations from the mean (mean ± S.D. = 0.094 ± 0.53) suggest a strong interaction between that gene and Lcn2. 49 genes meet this criterion. (C) Volcano plot summarizing the calculated P values of each log10 insertion read count difference counts between Lcn2+/+ and Lcn2-/- mice. The dotted line (significant P value cutoff = 1.3×10^−5^) indicates the threshold for consideration of a P value as significant after correction for multiple comparisons. 43 of 49 genes with log10 insertion read count ratios greater than or less than 3 standard deviations from the mean have a P value < 1.3×10^−5^, including gltA, which is shown in black. (D) Locations and frequencies of transposon insertions in gltA. (E) WT KPPR1, KPPR1ΔgltA, and KPPR1ΔgltApGltA were grown in LB (n = 3, mean displayed ± SEM). (F) Area under curve analysis of WT KPPR1, KPPR1ΔgltA, and KPPR1ΔgltApGltA growth in LB (n = 3, Tukey’s multiple comparison test following ANOVA, mean displayed ± SEM). (TIF) [file ppat.1008010.s001.tif]

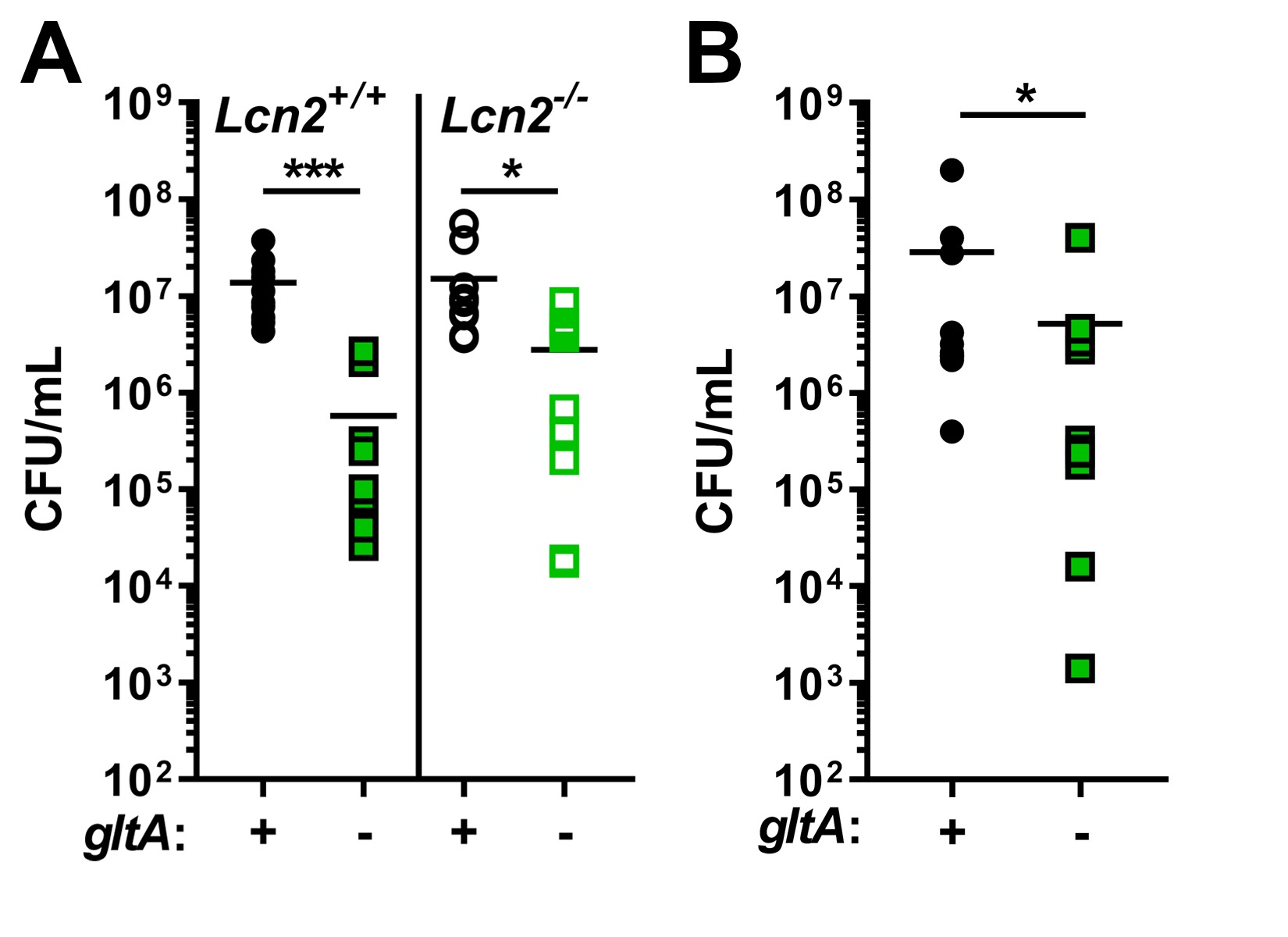

Supplement: S2 Fig — (A) C57BL/6J mice or isogenic Lcn2-/- mice were retropharyngeally inoculated with approximately 1×10^6^ CFU of a 1:1 mix of WT KPPR1 and KPPR1ΔgltA and lung bacterial burden was measured after 24 hours (n = 10 per group, mean displayed, *P < 0.05, ***P < 0.0005, Student’s t test). (B) C57BL/6J mice were retropharyngeally inoculated with approximately 1×10^6^ CFU of either WT KPPR1 or KPPR1ΔgltA and lung bacterial burden was measured after 24 hours (n = 10 per group, mean displayed, *P < 0.05, Student’s t test). (TIF) [file ppat.1008010.s002.tif]

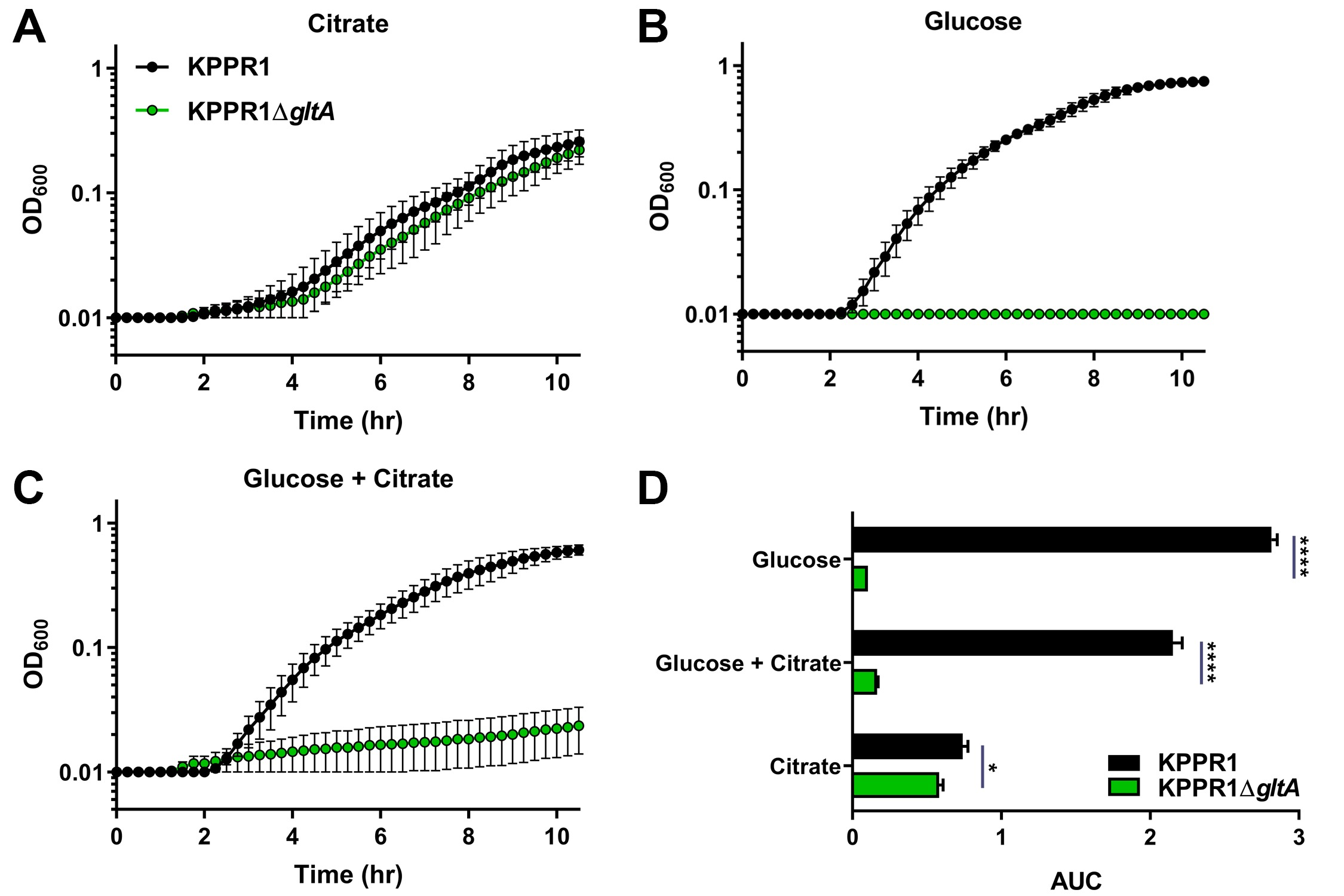

Supplement: S3 Fig — WT KPPR1 and KPPR1ΔgltA were grown in M9 minimal media with (A) 10 mM citrate, (B) 0.4% glucose, or (C) 0.4% glucose and 10 mM citrate (n = 3, mean displayed ± SEM). (D) AUC analysis of WT KPPR1 and KPPR1ΔgltA grown in M9 minimal media with 10 mM citrate, 0.4% glucose, or 0.4% glucose and 10 mM citrate (n = 3, ***P < 0.0005, ****P < 0.00005, Student’s t test, mean displayed ± SEM). (TIF) [file ppat.1008010.s003.tif]

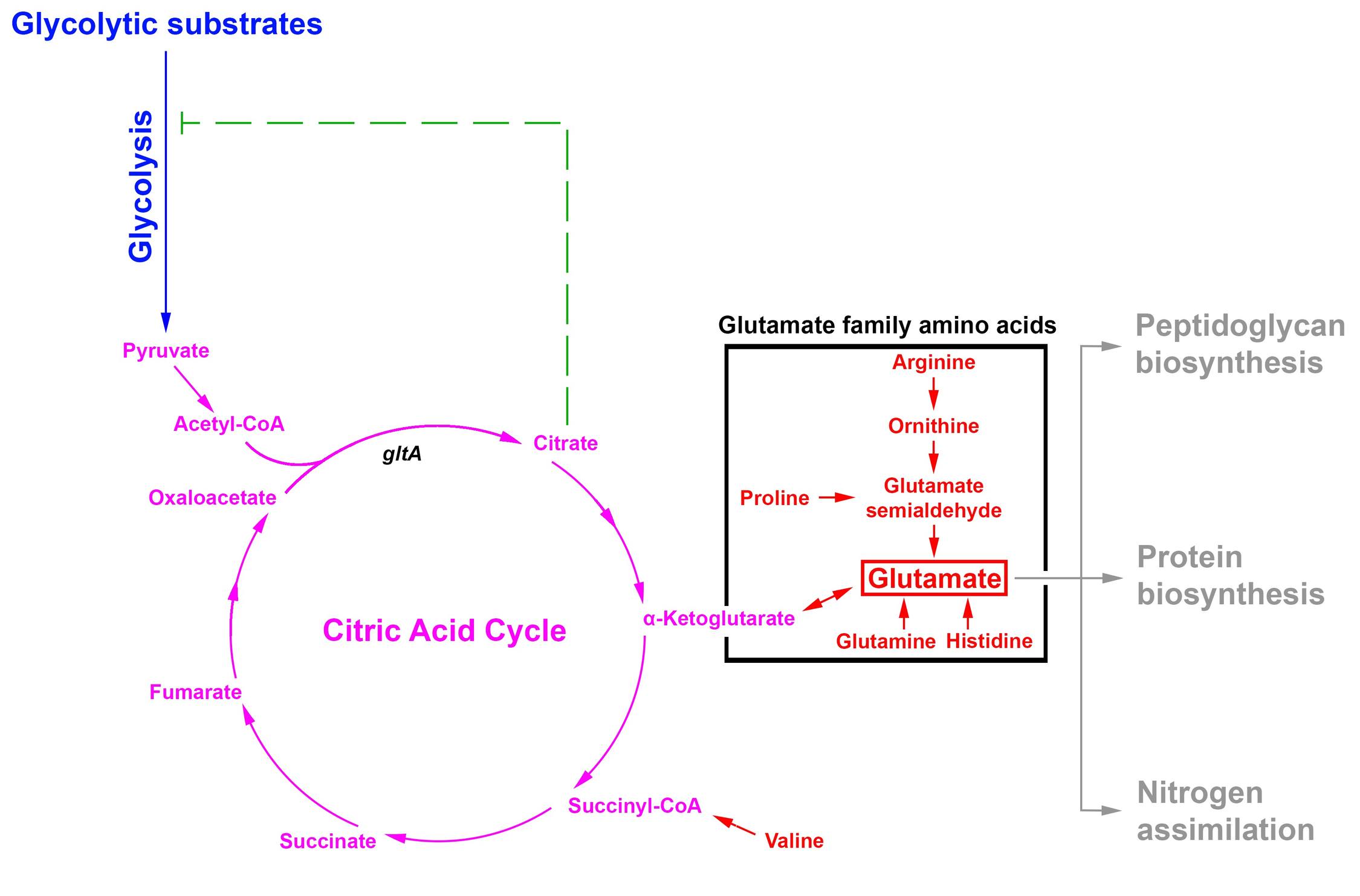

Supplement: S4 Fig — Glutamate family amino acids enter the citric acid cycle via α-ketoglutarate and exit the citric acid cycle for protein and peptidoglycan biosynthesis or nitrogen assimilation after conversion to glutamate. Glutamate plays a central role in these processes due to its position as a hub of multiple metabolic pathways. Deletion of gltA inhibits the ability of Kp to use metabolic substrates outside of the glutamate family of amino acids, resulting in significantly less metabolic flexibility. (TIF) [file ppat.1008010.s004.tif]

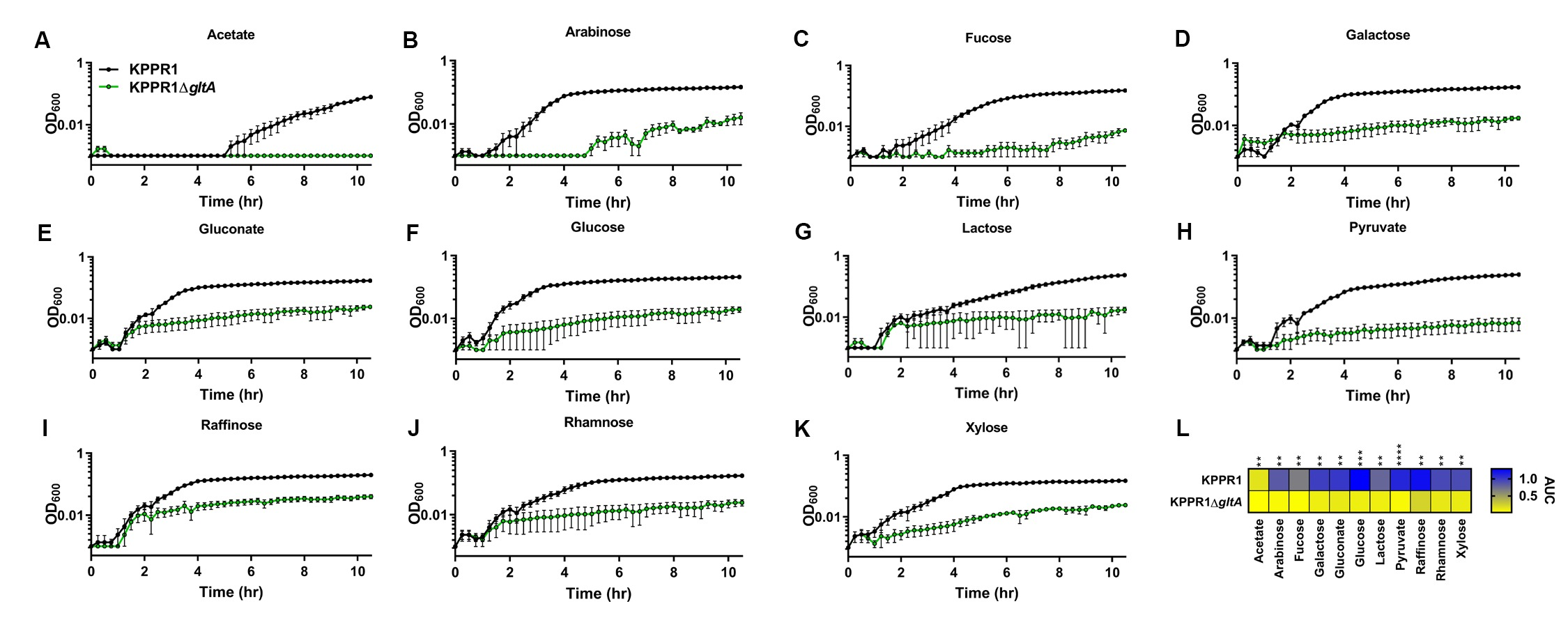

Supplement: S5 Fig — WT KPPR1 and KPPR1ΔgltA were grown in M9 minimal media + 5 mg/mL of (A) acetate, (B) arabinose, (C) fucose, (D) galactose, (E), gluconate, (F) glucose, (G) lactose, (H) pyruvate, (I) raffinose, (J) rhamnose, or (K) xylose (n = 3, mean displayed ± SEM). (L) AUC analysis of WT KPPR1 and KPPR1ΔgltA growth in M9 minimal media + 5 mg/mL specific sugar (n = 3, **P < 0.005, ***P < 0.005, Student’s t-test, mean displayed ± SEM). (TIF) [file ppat.1008010.s005.tif]

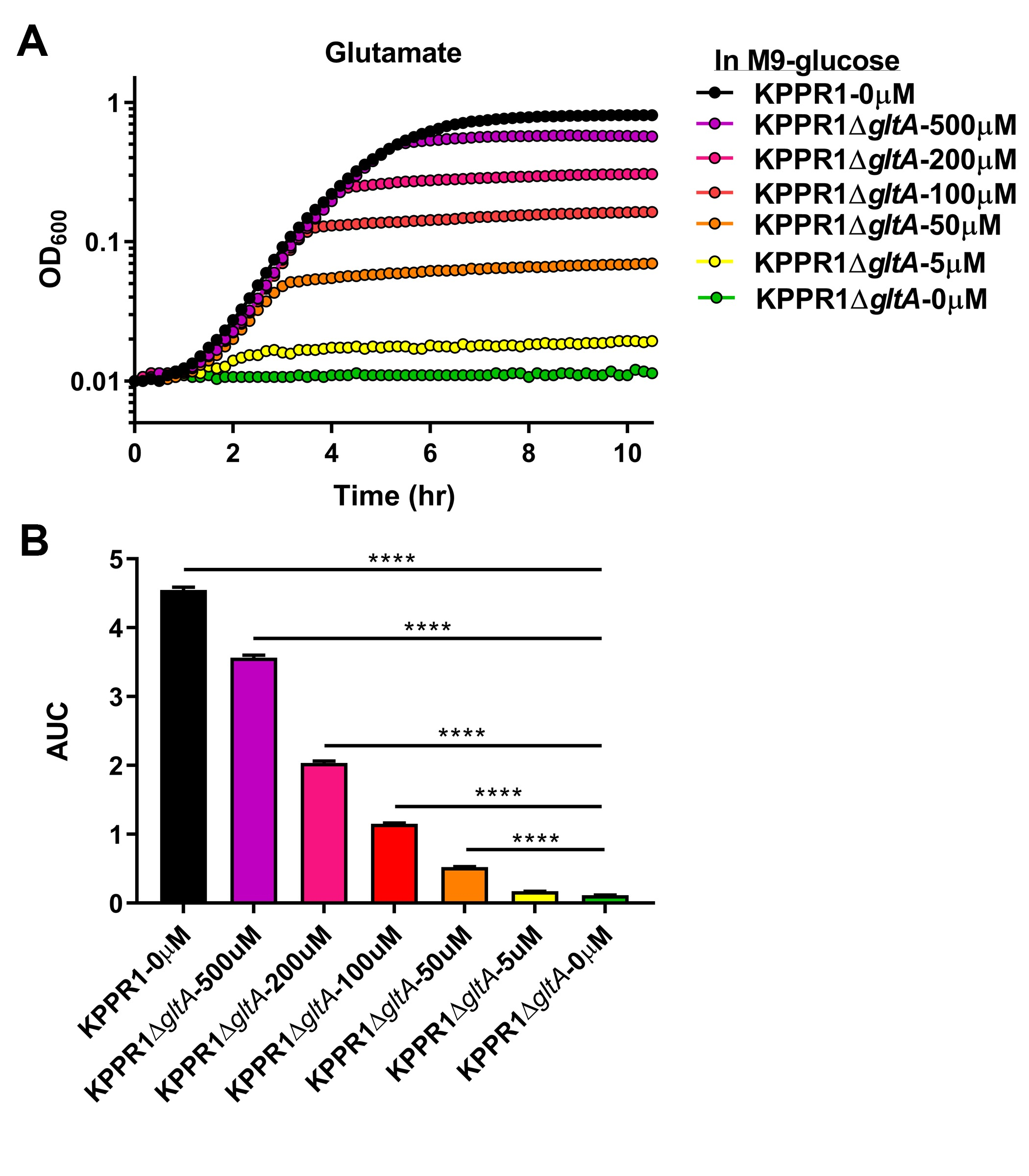

Supplement: S6 Fig — (A) WT KPPR1 and KPPR1ΔgltA were grown in M9 minimal media + 0.4% glucose with increasing concentrations of glutamate (n = 3, mean displayed ± SEM). (B) AUC analysis of WT KPPR1 and KPPR1ΔgltA were grown in M9 minimal media + 0.4% glucose with increasing concentrations of glutamate (n = 3, ****P < 0.00005, Tukey’s multiple comparison test following ANOVA, mean displayed ± SEM). (TIF) [file ppat.1008010.s006.tif]

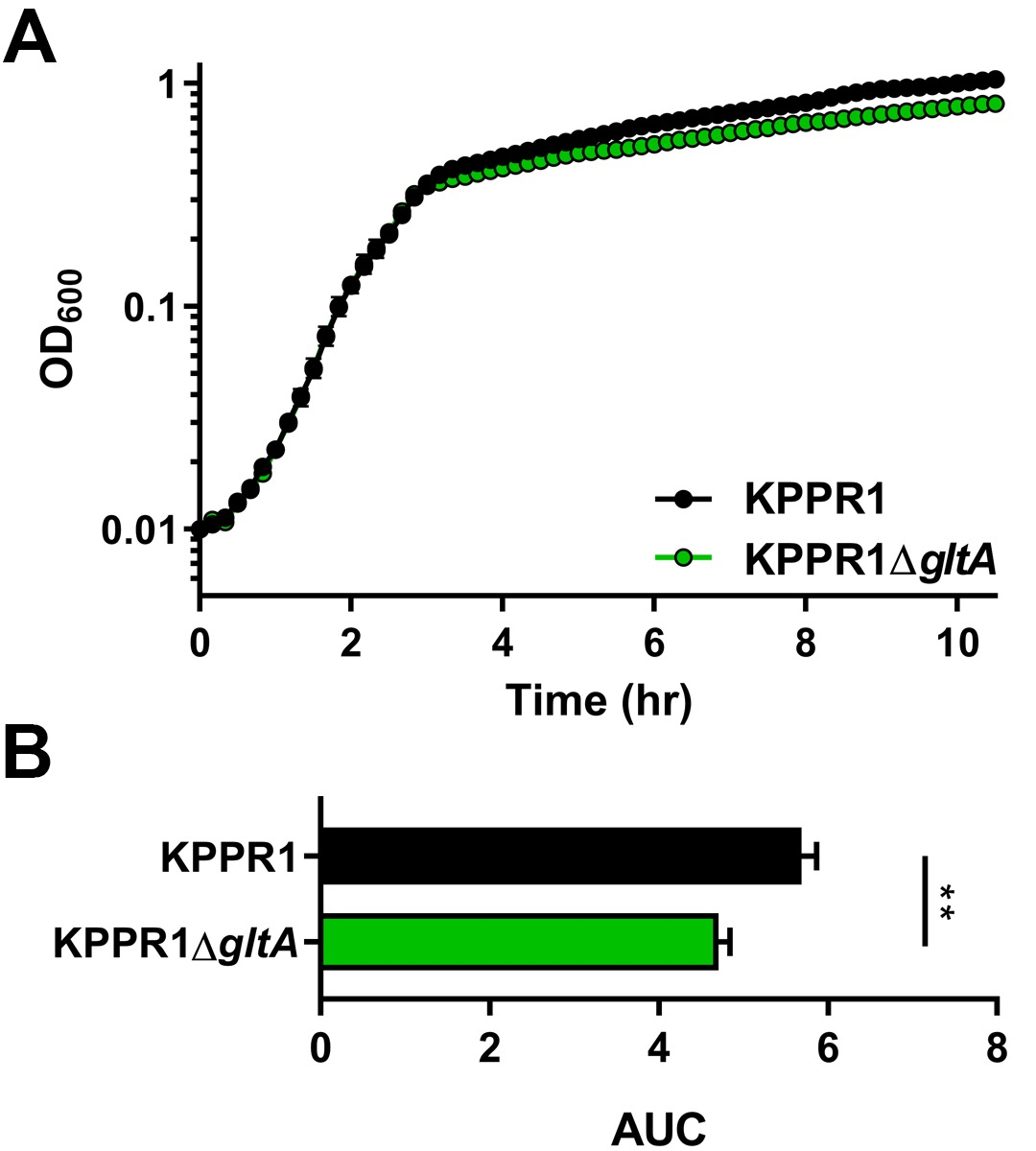

Supplement: S7 Fig — (A) WT KPPR1 and KPPR1ΔgltA were grown in M9 minimal media + 20% non-heat-inactivated murine serum (n = 3, mean displayed ± SEM). (B) AUC analysis of WT KPPR1 and KPPR1ΔgltA growth in M9 minimal media + 20% non-heat-inactivated murine serum (n = 3, **P < 0.005, Student’s t-test, mean displayed ± SEM). (TIF) [file ppat.1008010.s007.tif]

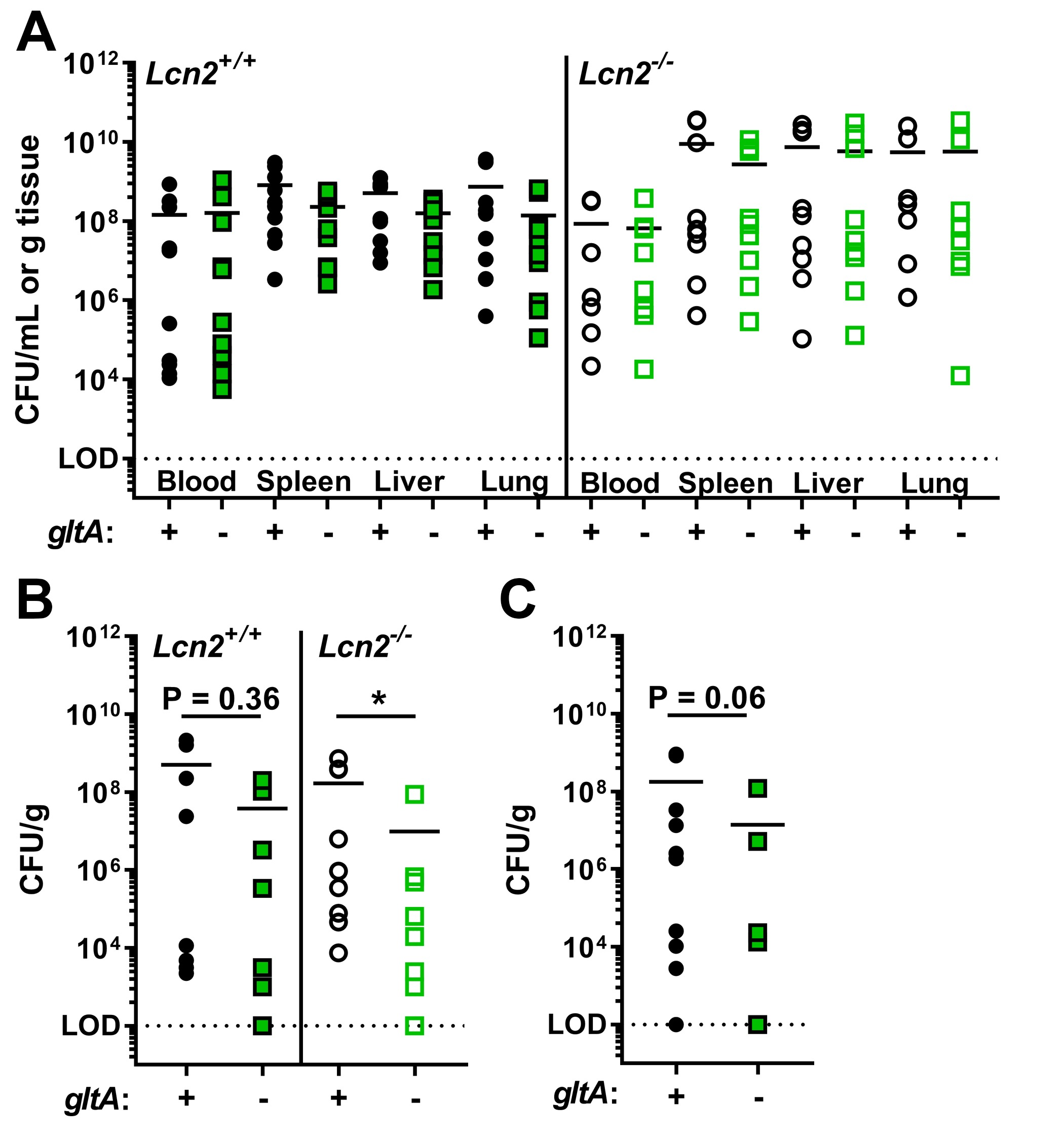

Supplement: S8 Fig — (A) C57BL/6J mice or isogenic Lcn2-/- mice were intraperitoneally inoculated with approximately 1×10^6^ CFU of a 1:1 mix of WT KPPR1 and KPPR1ΔgltA and bacterial burden was measured after 24 hours (n = 9–11 per group, mean displayed, *P < 0.05, Student’s t test). (B) C57BL/6J mice or isogenic Lcn2-/- mice were orally inoculated with approximately 5×10^6^ CFU of a 1:1 mix of WT KPPR1 or KPPR1ΔgltA and cecal bacterial burden was measured after 48 hours (n = 8–9 per group, mean displayed, Mann-Whitney test). (C) C57BL/6J mice were orally inoculated with approximately 1×10^6^ CFU of WT KPPR1 or KPPR1ΔgltA and cecal bacterial burden was measured after 48 hours (n = 9–10 per group, mean displayed, Mann-Whitney test). (TIF) [file ppat.1008010.s008.tif]

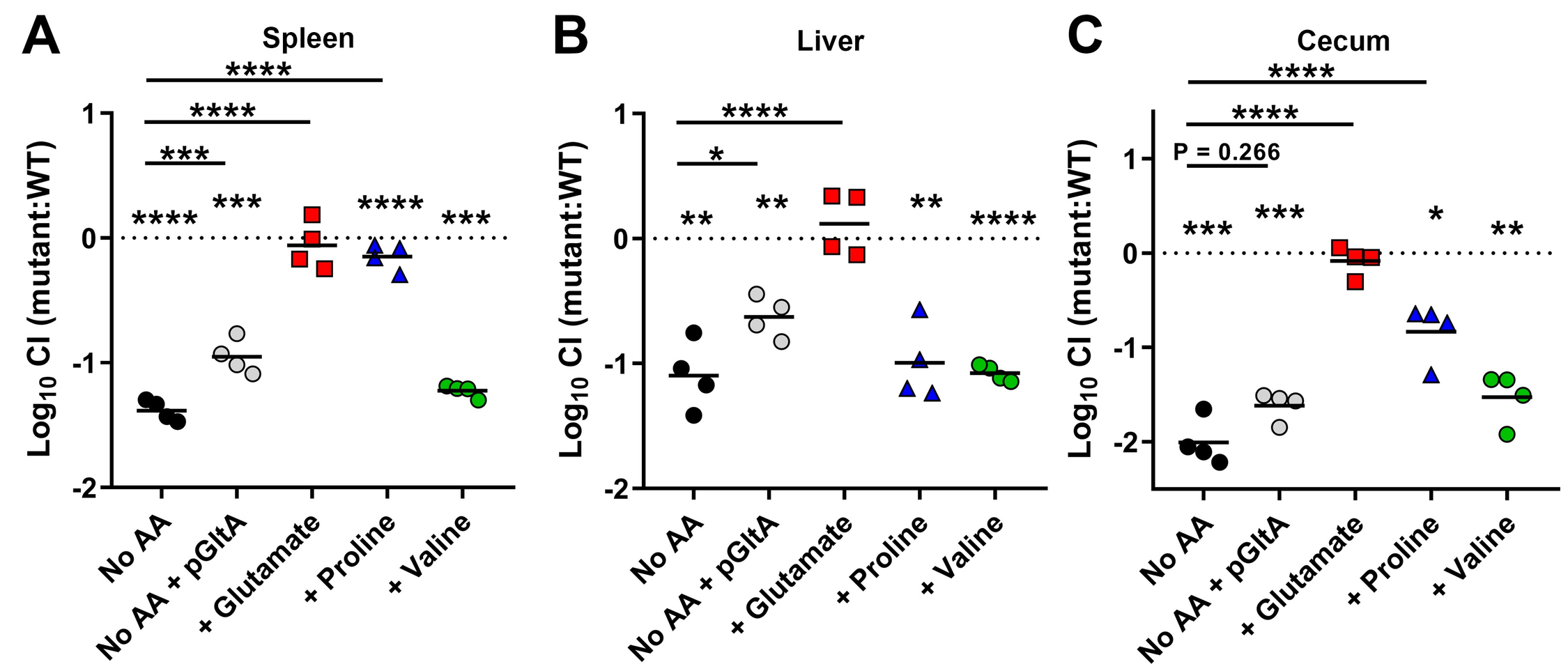

Supplement: S9 Fig — Murine (A) spleen, (B) liver, and (C) cecum homogenate generated from uninfected C57BL/6J mice with or without amino acids was inoculated with a 1:1 mix of WT KPPR1 and KPPR1ΔgltA or WT KPPR1 and KPPR1ΔgltApGltA. Bacterial burden was measured after 24 hours, and log10 competitive index of the mutant strain compared to the WT strain was calculated for each sample (n = 4 mice per group, *P < 0.05, **P < 0.005, ***P < 0.0005, ****P < 0.00005, one-sample t test or Tukey’s multiple comparison test following ANOVA). (TIF) [file ppat.1008010.s009.tif]
